# Heat, health and inequalities in the WHO European region – a scoping review with an intersectional lens

**DOI:** 10.1186/s12939-026-02787-1

**Published:** 2026-02-23

**Authors:** Katharina J. Pascale Wabnitz, Lilly Leppmeier, Romy Kuempfel, Anna Leibinger, Enno Nowossadeck, Eva Rehfuess, Kerstin Sell, Bernice Yanful, Karin Geffert

**Affiliations:** 1 Chair of Public Health and Health Services Research, Institute of Medical Information Processing, Biometry and Epidemiology, Faculty of Medicine, Ludwig-Maximilians-Universitaet Munich, Munich, Germany; 2 Pettenkofer School of Public Health, Munich, Germany; 3 Institute for Ethics and History of Health in Society, Faculty of Medicine, University of Augsburg, Augsburg, Germany; 4 Department of Epidemiology and Health Monitoring, Robert Koch Institute, Berlin, Germany; 5 Department of Sociology, Toronto Metropolitan University, Toronto, Canada

**Keywords:** Climate change, Heat, Health, Equality, Equity, Intersectionality, WHO European region

## Abstract

**Background:**

Climate change exerts diverse impacts on human health, with heatwaves emerging as a substantial concern. Social and health inequalities play a decisive role in this. Older people, children, and people experiencing homelessness or with low socioeconomic status, among others, are particularly affected by the health effects of heat and heatwaves. There is little evidence on the burden of being affected by multiple determinants of inequalities in the context of heat events. We aimed to map the research evidence on inequalities in heat-related health outcomes and their determinants in the World Health Organization (WHO) European region, applying an intersectional lens. This means considering how interlocking systems of power and oppression interact to affect the health and wellbeing of people differentially based on their varying and diverse social positions and identities.

**Methods:**

To this end, we undertook a scoping review of reviews. In 7/2023 and 8/2023, we systematically searched Pubmed and Epistemonikos and updated our searches in 9/2025. We mapped included studies based on methodology and determinants of inequalities considered in the analysis of heat impacts on health.

**Results:**

We screened 968 unique records and ultimately included 28 reviews. Included reviews cover all parts of Europe. Age and sex/gender were the most frequently assessed determinants of inequalities in this body of evidence. Only one meta-analysis presented disaggregated data for subgroups. Intersectionality or related terms were only explicitly mentioned in four reviews, and no review applied intersectionality as a foundational paradigm.

**Discussion:**

The findings suggest that age and sex/gender are more broadly assessed in the literature on heat-related health outcomes than other determinants. Whether authors assessed sex or gender is mostly not explicitly stated, impeding categorization as either a structural (gender) or intermediary (sex) determinant. Overall, intermediary determinants were more frequently assessed than structural determinants. Less frequently assessed determinants such as ability, income or education require further investigation in terms of their effects on health outcomes, as well as their amenability through generic or tailored measures. Intersectionality is only minimally reflected in the included literature. Future studies should employ mixed-methods approaches that seek to not only quantify heat-related health inequalities but also establish why they arise and whether and to what extent they are policy-amenable.

**Supplementary Information:**

The online version contains supplementary material available at 10.1186/s12939-026-02787-1.

## Background

Climate change represents one of the greatest current challenges to human health and well-being [1]. As a result of climate change, extreme weather events, such as heat waves, heavy rain, drought, coastal flooding or wildfires, will occur more frequently worldwide, including in Europe [2, 3]. Mounting evidence shows that there is already an increase in the duration, intensity and frequency of heat waves globally and in Europe [4]. This trend of an increase in the number of heat waves as well as the maximum heat wave temperature is expected to continue in the future [5]. Europe is the fastest warming continent in global comparison with several climate risks already having reached critical thresholds according to recent assessments [6]. These include megadroughts affecting large regions over years, extreme precipitation, coastal floods and storm surges due to sea-level rise as well as heatwaves. Hence, the health impacts from climate change which include health-related conditions, food insecurity and vector-borne diseases are escalating in scale, complexity and interconnectedness in the WHO European region [7].

Heat poses a risk to human health [8, 9]. In 2022, heat-related mortality was estimated at 61,672 deaths (95% CI = 37,643–86,807) in Europe. Both exposure to heat as well as heat-related health outcomes are unequally distributed [10]. For instance, factors such as old and very young age, chronic diseases, low socio-economic status, occupational status and homelessness tend to be associated with elevated exposure and worse health outcomes in the context of heat and heat waves [2]. Thus, climate change and its consequences contribute to and worsen health inequalities [3].

Based on an understanding of health and disease as socially produced, the WHO Commission on the Social Determinants of Health (CSDH) distinguishes social causes of health, such as housing and physical environment and the social factors that determine the distribution of these causes, such as the socio-economic political context and power [11]. In their conceptual framework for action on the social determinants of health, social causes are conceptualized as intermediary determinants of health and distributing factors as structural determinants of health inequities. The framework distinguishes between gender as socially constructed and sex as biologically determined [11, 12]. Gender is therefore conceptualized as a structural determinant of health inequities and an important characteristic of an individual’s socioeconomic position whereas sex is conceptualized as an intermediary determinant of health. Sex and gender tend to be conflated in (public) health research, with “an essentialist, binary understanding of gender” being applied particularly in quantitative epidemiological analyses [12]. Different terms to refer to differences in health status are being used, sometimes also reflecting regional differences. For the purposes of this review, we chose to apply the following definitions: ‘Health inequalities’ refer to differences in health status between social groups which are due to chance, inevitable, or irremediable [13]. We use inequity to refer to systematic and modifiable differences between social groups which are considered unfair, evitable and remediable [13–15]. Arguably, the notion of inevitability is dynamic in that the understanding and appreciation of certain disease processes as well as the capability to prevent or treat certain diseases can change [16]. Furthermore, what is treatable, preventable or avoidable is subject to socio-political prioritization, negotiation and normative reasoning. Throughout this paper, we use the term inequality when we refer to any differences in exposure, susceptibility, social positions or health outcomes without further discussion of their evitability.

Intersectionality is understood as an analytical approach, a concept and also a movement which originated in black, feminist thinking [17]. It recognizes that the health and wellbeing of people are affected differentially by interlocking systems of power and oppression according to their diverse and overlapping social positions and identities [18–20]. These include, but are not limited to, ethnicity, gender, class or sexual orientation. Applying an intersectional lens to the assessment and alleviation of inequalities between population groups seeks to ground this work in an understanding of the “events and conditions of social and political life and the self [...] to be shaped by many factors in diverse and mutually influencing ways” [20]. Carrying out research based on this premise requires a focus on the joint effects of multiple axes of inequalities and their underlying determinants. It further necessitates reflexive work on the part of the researchers to position themselves and their work vis à vis those joint effects and their determinants.

Differential social positions can lead to differential exposures to heat and other environmental factors which is a result of power imbalances and oppression. Consequently, the type and severity of the health effects experienced due to multifactorial exposures can vary, as do the consequences of illness [21]. For health-related impacts of climate change, concepts of climate justice from a public health perspective [22] or concepts of environmental justice in public health research [23], stating that “an EJ [environmental justice] study will consider the intersection of social disadvantage with environmental factors for the ultimate goal of achieving health equity”, have been discussed in the literature. An intersectional lens can offer a deeper understanding of how different population groups are differentially impacted by heat and heat exposure.

It is unclear to what extent the impact of heat on health, as a direct effect of climate change, has been investigated with an intersectional lens.

## Research aims

Our aim was to map the peer-reviewed research evidence on inequalities in heat-related health outcomes and their determinants in the WHO European Region with an intersectional lens.

## Methods

### Study design and rationale

We conducted a scoping review of reviews and initially also of recent primary studies (see deviation from the study protocol). Scoping reviews enable researchers to collate the types and volume of scientific studies available in a given field, to identify existing knowledge gaps and to investigate epistemological approaches to research on a particular topic [24, 25]. To keep the review manageable, yet to provide a comprehensive overview of the available evidence, we initially decided to apply a two-pronged approach. As outlined in detail in our prospectively registered protocol [26], we searched for (i) reviews published over the last 20 years and (ii) primary studies published in the year before project inception. We followed recommendations in the Preferred Reporting Items for Systematic reviews and Meta-Analyses extension for Scoping Reviews extension for Scoping Reviews (PRISMA-ScR) to report our methods and results [27].

### Deviation from the study protocol

We deviated from the published protocol in that we decided not to map studies onto the framework by Diderichsen and Hallqvist [28]. We found that, contrary to our initial assessment, it did not lend itself well to discussing to what extent identified inequalities might be avoidable (i.e. inequities). We do elaborate on this aspect in the discussion. We also did not further investigate the structures and actors that play a role in generating or addressing health inequities, as we were not able to extract enough meaningful information from the data.

We also reran our searches for review papers during peer review and therefore decided to move the second part of our review – the analysis of primary studies – to the Appendix as we consider those covered by our updated searches for reviews (Appendix 5 and 6).

### Eligibility

We included peer-reviewed articles that reported heat-related health outcomes in the WHO European region and provided outcomes disaggregated for stratifiers as per the PROGRESS-Plus framework^1^ (Table 1) [29]. The WHO European Region covers 53 countries from Europe to Central Asia [30].

**Table 1:** Inclusion and exclusion criteria for reviews

Population	Inclusion criteria	Exclusion criteria
	Population or population groups in the WHO European region	Any population(s) residing outside the WHO European region; athletes as a vulnerable population
Exposure	High ambient temperatures, including indoor temperature, heat, heat waves	Any other exposure, including mixed exposures e.g. heat and air pollution
Inequalities in health outcomes and their determinants	Inequalities in health outcomes (i.e. any measure of mortality or morbidity) and their structural and intermediary determinants	Reporting of aggregated outcomes without further disaggregation Indirect health effects (e.g. biochemical markers; epigenetic factors) and other non-health effects such as loss of productivity
Publication type	Articles published in peer-reviewed academic journals or on pre-print servers	Articles published in any other media, including books, book chapters, and any other grey literature
Study design	Reviews (e.g. systematic reviews, scoping reviews) based on systematic searches	Prospective modelling studies and any non-empirical publications, such as editorials, essays, commentaries, or blog posts
Language	English and German	Any other language
Publication date	01/01/2003–01/09/25	Published before or after this period

### Identification of studies

Our search strings were built around three concepts: Heat AND Health AND WHO European region. For the full search strings, see Appendices 1 and 2. The initial search strategy was adapted from an existing systematic review [31]. Additionally, we included further search terms from a concurrent systematic review on exposure to heat of different population groups [32]. We searched PubMed on 24/07/2023 employing our search string supplemented with filters to include only meta-analyses, reviews or systematic reviews; we then searched Epistemonikos on 03/08/2023 using an adapted search string. For capacity reasons, we did not conduct backward or forward citation searches.

We reran the searches for systematic reviews on 26/09/2025, using the same search strings and databases as described above.

### Data collection

#### Selection of studies

After import of search results into the reference management software Endnote [33] and de-duplication, two authors (LL and KW) independently conducted title and abstract screening of the same 20% of records, followed by discussion of unclear cases to calibrate the screening process. Where no agreement was obtained, further authors were consulted. The remaining records were screened by one author (LL) and unclear cases were discussed between two authors (LL and KW). At the full text screening stage, 20% of the studies were double screened (LL and KG) to ensure consistent application of eligibility criteria, followed by discussion of unclear cases. Reasons for exclusion of full texts were documented. The collaborative web application Rayyan [34] was used for both title and abstract and full text screening of the retrieved records.

The same procedure was applied to the review update (KG und KW).

### Data extraction

We developed a data extraction sheet in Microsoft Excel (Appendix 3). A priori identified categories for data extraction included study characteristics (e.g. authors, title), study information (e.g. population, exposure measure, outcome measure) and health outcomes, including determinants of inequalities. Three authors (LL, KW and KG) pilot-tested, refined and added categories iteratively by extracting data from 5 reviews each and comparing and discussing their results. Categories in the final extraction sheets included study characteristics, outcome and exposures measures and determinants of inequalities assessed. For the reviews, all determinants presented from included studies in results or discussions of the review were extracted. Data extraction of the remaining studies was carried out by one author (KW, LL or KG) and checked by a second author (KW, LL or KG).

The same procedure was applied to the review update (KG und KW).

### Analysis

#### Mapping of determinants of inequalities in heat-related health outcomes

Determinants of inequalities assessed in studies were extracted and grouped according to the Cochrane PROGRESS-Plus framework [29] and the CSDH conceptual framework for action on the social determinants of health [11] and displayed tabularly. The number of studies that assessed each determinant was calculated.

#### Assessing and applying intersectionality

We chose a two-tiered approach to analyzing intersectionality: First, we sought to examine whether and how included studies drew on this approach. To this end, we searched each paper for the term ‚intersect*‘. For research that applied an intersectional lens, we extracted the methods the authors used to operationalize this approach. We also assessed whether authors presented a reflexivity statement.

Second, we took an intersectional approach to this review project by engaging in reflection on our own positionality and by discussing our results in light of our background research on intersectionality theory. Moreover, we consulted with an expert (BY who is also an author of this paper) in intersectionality theory and practice from the Canadian National Collaborating Centre for Determinants of Health (NCCDH) [17] to develop the methodological approach as we were not able to identify any published guidance on how to apply an intersectional lens to evidence synthesis at the time of carrying out this research. Discussion papers and guidance we consulted at the time of developing the protocol focused on implications of intersectionality for qualitative research and in recent contributions also on quantitative research [35–38]. Following various rounds of discussion within the author team, we settled on the approach described above and elaborate on it in the discussion.

#### Reporting of results

We summarized and reported the data narratively and in tabular form.

### Reflexivity statement

In order to uphold the core tenets of intersectionality theory, specifically the principle of reflexivity, we will provide some insights into our team’s positionality and the broader context in which this work was conducted. We (the authors) are affiliated to several different institutions and the team brought a diverse range of experiences in relation to the topic of this study to the project. The researchers have a background in medicine (KW, KS, KG) and/or public health and epidemiology (LL, KW, AL, KS, KG, ER, RK, ES). This project was commissioned by the Robert Koch Institute (RKI), Germany’s national public health authority, with the aim to inform their work on the social determinants of health in the context of a changing climate and environment.

We understand health and wellbeing to be politically determined through the socially patterned environments and living conditions in which people are born, live, study and work. We strive for our own works as public health researchers and practitioners to be carried out according to highest scientific standards and to be relevant for (political) decision-makers.

We did not have any prior experience in adopting an intersectional lens to research. We have not involved representatives of population groups who are potentially vulnerable to heat waves in this research. We deliberately sought to not preemptively narrow our findings by including population groups known to be heat-sensitive in the search string. Following from this rationale, we could only have usefully involved them after having screened the retrieved studies and extracted the data. Given that the narrow time frame and limited human resources for this project would not have allowed for any iterations of the searches and that development of recommendations was out of scope for this commissioned work, we refrained from including further perspectives in the research. We did however consult with an expert on intersectionality from the Canadian NCCDH to discuss and critically reflect on our approach to adopting an intersectionality lens for this project.

As researchers, we are aware that our individual social positions, values, worldviews and convictions shape our interpretation of the data. We acknowledge that those aspects may introduce both conscious and unconscious biases into the research process. We actively engaged in self-reflection throughout this study to minimize the impact of these biases.

## Results

### Results of searches

**Fig. 1:**
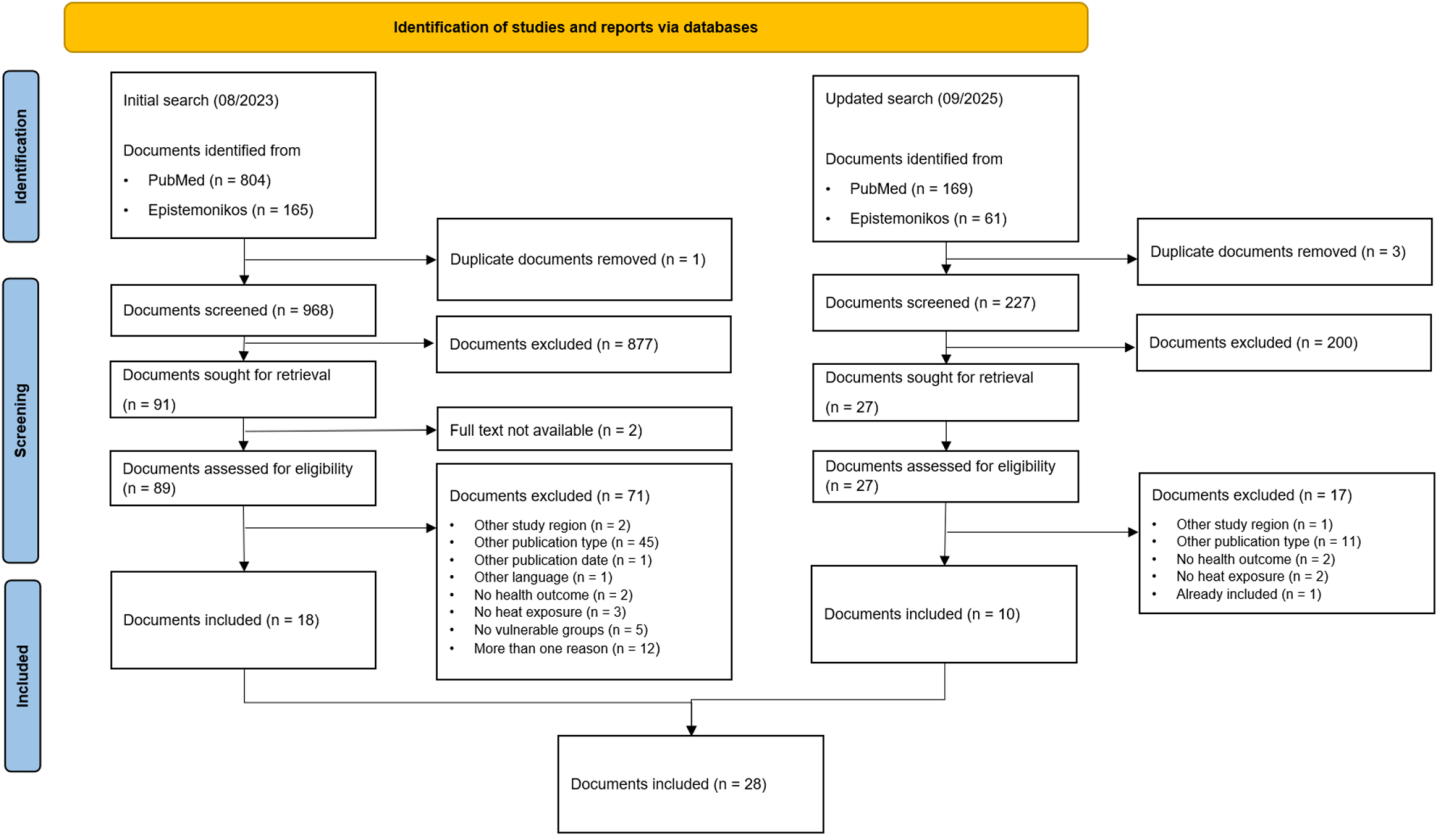
PRISMA-ScR Flow Chart (PS = primary study, R = review)

After de-duplication, initial database searches yielded 968 unique records (Fig. 1). Overall, 89 reviews were included for full text review after title and abstract screening. Of these, 18 reviews were included. Our updated searches for reviews yielded 227 unique records, of which 27 were included for full text review after title and abstract screening. After reviewing the full texts, of those, 10 reviews were added to the study, yielding a total of 28 included reviews.

### Description of included studies

#### Study design

Of the 28 included reviews, we identified eight systematic reviews with a meta-analysis [39–46], seven systematic reviews without a meta-analysis [47–53], seven scoping reviews [54–60] and six reviews with narrative synthesis [61–66]. Overall, 12 reviews reported an assessment of the quality of their included studies [40, 43, 44, 46, 47, 49, 51–53, 55, 58, 59]. One of the systematic reviews without meta-analysis did not retrieve any studies, thus representing an empty review [47].

#### Geographic focus

Most reviews (*n* = 16) included data from all over the world, including the WHO European region [39, 40, 42–48, 50, 53, 57–59, 64, 65]. Two reviews focused on European countries [51, 66], one on North America and Europe [54], one on the arctic and subarctic region [49], and one on the Mediterranean basin [41]. Two reviews focused on one country only (Italy [55], Ireland [56]), three on the United Kingdom (UK) [52, 61, 62], and two on Germany [60, 63]).

#### Population

15 reviews did not focus on any specific sub-population [39, 41, 46, 47, 49, 53–55, 57, 60–65], two were concerned with workers [40, 50], two with populations affected by chronic lung disease [42, 45], two with the elderly population [43, 66] and two with embryos and fetuses [44, 48]. One review each focused on the “population affected by extreme weather events” [52], patients admitted to the emergency department [59], women aged 15 – 49 [58] and “populations vulnerable to heat” [56].

Details can be found in Appendix 4.

### Mapping of determinants of inequalities in heat-related health outcomes

We identified 12 categories for determinants of health inequalities: sex/gender; age; marital status/social network; ethnicity; education; income; work including employment status; area of living and housing conditions (including infrastructural and social aspects such as population density); environmental parameters (including aspects such as vegetation index in the neighborhood); health conditions including ability; compound indicators of socio-economic status (including different socio-economic status indicators such as poverty indicators or deprivation indices); other (for example parity).

Most reviews included disaggregated outcomes for age (*n* = 25) and sex/gender (*n* = 19) (Table 2). None of the included studies made their operationalization of sex/gender explicit. All of them used binary categories such as male/female, men/women and the terms sex and gender were only applied correctly as per the definitions in the introduction by few authors, e.g. Zhou and colleagues [46]. Therefore, all studies that assessed sex/gender were grouped in one category (*n* = 19) (Table 2).

Disaggregated outcomes for area of living/housing conditions were reported in 12 reviews, e.g. differentiation between urban and rural areas [39, 41, 49, 51, 52, 56, 57, 63, 65], but also nursing home residency [59, 61, 62]. Further reviews disaggregated outcomes for the determinants ethnicity (*n* = 9) [40, 41, 50, 54, 56, 57, 59, 65, 66] and health condition/ability (*n* = 14) [42, 45, 46, 49, 51, 52, 54–57, 59–61, 65]. Within these, risky health behaviors such as smoking were mentioned in two reviews [51, 54]; alcohol or drug abuse were included in three reviews [52, 59, 61].

Aspects that can be broadly categorized as socioeconomic status, excluding income and education, were mentioned in 13 reviews [39, 45, 46, 51, 52, 54, 56, 57, 59, 62, 64–66]. Of those, only four made explicit how socioeconomic status was operationalised [39, 57, 62, 65].

For other determinants of inequalities, namely marital status/social network, education, income, work, ecological parameters, disaggregated health outcomes were included in fewer than 10 reviews for each determinant (Table 2).

One systematic review with a meta-analysis presented disaggregated health outcomes for subgroups regarding the determinants of sex/gender, age and work [40]. Another systematic review only mentioned further subgroup analysis within the elderly in some of the included studies but these were not presented [53]. Some of the narrative reviews reported studies which analyzed disaggregated risks of subgroups e.g. the higher risk for women over 65 years compared to men [66] or the higher risk of persons with chronic psychiatric conditions under 65 years compared to those above [62]. None of the included studies assessed intersecting determinants of inequalities.

**Table 2:** Determinants of inequalities considered in reviews

	Determinants of inequalities considered in reviews											
		Structural					Intermediary					
**Study ID**	**other**	**ethnicity**	**education**	**income**	work*	**socioeconomic status**	**area of living/housing conditions**	**health condition including ability**	**ecological parameters**	**marital status/social network**	**age**	**sex/gender**
Systematic Review with meta-analysis												
Alentorn_2023	nr	nr	nr	nr	nr	yes	yes	nr	yes	nr	nr	nr
Byun_2024	nr	nr	nr	nr	nr	nr	nr	nr	nr	nr	yes	nr
Fatima_2021	nr	yes	nr	nr	yes	nr	nr	nr	nr	nr	yes	yes
Krishnakumar_2024	nr	nr	nr	nr	nr	nr	nr	nr	nr	nr	yes	nr
Perry_2023	nr	yes	yes	nr	yes	nr	yes	nr	nr	nr	yes	yes
Tran_2025	nr	nr	nr	nr	nr	yes	nr	yes	nr	yes	yes	yes
Witt_2015	nr	nr	nr	nr	nr	nr	nr	yes	nr	nr	nr	nr
Zhou_2025	nr	nr	nr	nr	yes	yes	nr	yes	nr	yes	yes	yes
Systematic Review without meta-analysis												
Dickinson_2025	nr	nr	nr	nr	nr	yes	yes	yes	yes	nr	yes	yes
Gupta_2012	nr	nr	nr	nr	nr	nr	nr	nr	nr	nr	yes	nr
Haghighi_2021	nr	nr	nr	nr	nr	nr	nr	nr	nr	nr	yes	nr
Hedlund_2014	nr	nr	nr	nr	nr	nr	yes	yes	yes	nr	yes	yes
Levi_2018	nr	yes	nr	nr	yes	nr	nr	nr	nr	nr	yes	yes
Pantavou_2025	yes	nr	nr	nr	yes	nr	nr	nr	nr	nr	yes	yes
Weilnhamer_2021	nr	nr	nr	yes	nr	yes	yes	yes	yes	nr	yes	yes
Scoping Review												
Cicci_2022	nr	yes	nr	nr	nr	yes	nr	yes	yes	nr	yes	yes
Edwards_2025	nr	yes	nr	nr	nr	yes	yes	yes	nr	yes	yes	yes
Gebhardt 2023	nr	nr	nr	nr	nr	nr	nr	yes	nr	nr	yes	nr
Massazza_2022	nr	nr	nr	nr	nr	nr	nr	yes	nr	nr	yes	yes
Paterson_2020	nr	yes	nr	nr	yes	yes	yes	yes	nr	nr	yes	yes
Wu_2023	nr	yes	nr	yes	yes	yes	yes	yes	nr	nr	yes	yes
Narrative Review/ Literature Search												
Anderson_2013	nr	nr	nr	nr	nr	nr	yes	yes	nr	yes	yes	nr
Arbuthnott_2017	nr	nr	nr	nr	nr	yes	yes	nr	nr	nr	yes	yes
Bittner_2014	nr	nr	nr	nr	nr	nr	yes	nr	nr	nr	yes	nr
Green_2019	nr	nr	yes	nr	nr	yes	nr	nr	nr	nr	yes	yes
Martiello_2010	nr	yes	yes	yes	yes	yes	yes	yes	nr	yes	yes	yes
Meherali_2024	nr	nr	nr	nr	nr	nr	nr	nr	nr	nr	nr	yes
Van_Steen_2019	nr	yes	nr	yes	nr	yes	nr	nr	nr	yes	yes	yes

**Legend**: nr = not reported, *including employment status, (un)employment rate, working environment

### Assessment of intersectionality in included studies

Of all included studies, four reviews referred to intersectionality or related terms in the respective discussion sections in a way which suggests some understanding of intersectionality theory. Cicci, Maltby et al. discussed that “it is likely that the intersectionality between individual and environmental factors affects the vulnerability and adaptability of some populations” [54]. Zhou and colleagues stated that “intersecting factors like socioeconomic status and comorbidities also likely modify sex-specific heat vulnerability” [46]. Dickinson and colleagues concluded that “Tailored support and targeted interventions that recognise and address these intersecting vulnerabilities are critical to ensuring that no group is left behind in disaster preparedness and response” [52]. In their scoping review on heat impacts on mental health in Germany, Gebhardt and colleagues concluded that “there are currently no studies […] that examine the connection between climate change and specific sociodemographic or sociological factors and intersectional discrimination (i.e. the reinforcing effects of interdependent systems of discrimination such as patriarchy, capitalism, colonialism, ableism […] in terms of mental health” [60].

For completeness, Meherali et al. wrote that “The intersection of climate change and maternal health demanded proactive adaptation strategies […]” [58]. We interpret this choice of wording to not be informed by intersectionality theory.

No study was carried out with intersectionality as its foundational paradigm. None of the 28 included reviews contained a reflexivity statement by the authors.

## Discussion

### Principal findings

This scoping review of reviews provides an overview of evidence syntheses that examine inequalities in heat-related health outcomes and their determinants in the WHO European region. In the 28 included reviews, disaggregated health outcomes linked to heat were most frequently reported based on age and sex/gender, and to a lesser extent by determinants such as ethnicity, area of living and housing conditions or ecological parameters. Two studies referred to intersectionality or related terms, but no study drew on intersectionality as its foundational paradigm. Accordingly, the compounded health effects of heat in groups that experience intersecting disadvantages are not regularly assessed in the included literature, but inequalities were mostly assessed unidimensionally.

### Results in context

Gaining an understanding of the nature of various determinants of health inequalities is important to effectively address them. To do so, it is important to empirically assess and politically negotiate the degree of evitability or redressability of any given health inequality. We found that most studies included in this review assessed the effects of age and sex/gender on heat-related health outcomes. Outcomes disaggregated by employment status, income, marital status/social network or education were only addressed in a limited number of studies. This is likely due to data availability and ease of methodological processing.

We used a merged category of sex and gender, thereby not differentiating between gender as a structural and sex as an intermediary determinant as per the conceptual framework for action on the social determinants of health. All studies operationalized sex/gender as binary and none made a clear reference to definitions or concepts of sex/gender. No study provided information as to how the data about sex/gender was gathered. Some used the terms interchangeably, making clear categorization of the determinant as either structural or intermediary, impossible. While it was not part of the aims for this review to assess operationalization of sex/gender, it is noteworthy that sex/gender sensitivity seems to be low in the included body of evidence. This is in line with findings from a scoping review on intersectionality and sex/gender sensitivity in quantitative health research [12]. In order to effectively address sex/gender-related health inequalities in the context of heat, it is important to know which inequalities can be attributed to gender and sex, respectively, as well as to apply multidimensional concepts of sex/gender from study design to publication. This points to a need for awareness and education among researchers and users of evidence alike.

In light of the merged category of sex/gender, the results indicate that intermediary determinants of health are more often considered in the included studies than structural determinants. If authors had mostly used gender based on an understanding of gender as socially produced and also operationalized this variable in a non-binary manner, there would be a balance between intermediary and structural determinants. Given prior research evidence [12, 67], we would cautiously assume that most of the data gathered for primary studies in the included reviews were likely based on a binary understanding of sex, which would shift the balance even further towards intermediary determinants. Structural determinants of inequities in health therefore seem to be less regularly addressed in the included body of literature which could be due to lack of awareness or methodological difficulties in processing such data and lack of primary data.

For this review, we decided to not narrow our searches by including examples of so-called ‘vulnerable’ populations in our search string to not perpetuate essentialist views of vulnerable groups and to avoid relevant studies not being retrieved. While it was out of our scope to discuss underpinning conceptions of vulnerability here, based on our results we would argue that current research on health effects of heat has rarely been grounded in an understanding of structural vulnerability, i.e. vulnerability to be the result of an individual’s position in a “hierarchical social order and its diverse networks of power relationships and effects” [68].

Overall, it was interesting to note that inequalities resulting from exposure to work environments and health-related harmful behaviors, such as high alcohol or illicit drug consumption, were only assessed in very few studies. Similarly, ability was rarely assessed. This points to gaps in the available literature regarding the health impacts of heat on certain population groups. A recently published systematic review without a meta-analysis assessing “social disadvantage on exposure to subjective and objective heat stress and related adaptive capacity” also found that more research on differences in health between advantaged and disadvantaged groups in the context of heat is needed [69]. In particular, the authors mentioned gaps in relation to occupational heat stress and heat stress arising from (informal) care work. They also concluded that the inequalities identified in their review are intersectional in nature and require intersectionality-informed approaches such as the intersectional climate justice framework by Amorim-Maia et al. [70], but they did not take an intersectional approach to their review.

### Intersectional lens – methodological considerations

We sought to carry out this review using an intersectional lens. This was operationalized (1) on the level of the analysis, i.e. by assessing whether and to what extent intersectionality was adopted as an analytical framework in the reviewed studies and (2) by engaging in reflexivity regarding our own motivations to carry out this research, underlying biases and assumptions and social identities. Hence, this review does not only map the available evidence in relation to heat, health and inequalities but also serves as an attempt to carry out an intersectionality-informed literature review which might inform future literature research that aims to examine and address health inequalities from an intersectional perspective.

Intersectionality, both as a paradigm or as an analytical approach is hardly reflected in the included body of evidence. Some studies employed methods deemed suitable to reflect intersectionality in quantitative analyses [38]. However, the application of certain methods alone cannot be considered an intersectional approach without explicitly grounding the research in intersectionality theory and an intention to reveal (and arguably address) disproportionate burdens in groups with intersecting social identities [37].

### Inequalities or inequities?

Establishing which (health) inequalities are to be considered inequities, i.e. are amenable to political, social or practical change and should be addressed as unjust, is a normative discussion [71]. As Vallgårda argued, judgements regarding individual or structural responsibility for health or other conditions fundamentally derive from views on human nature and amenability cannot easily be assessed based on a crude distinction between biological and social factors as this line is often blurred [16]. Whether and how scientific research can and should be carried out with a view to informing or even influencing normative and political discourse, is subject to current debates [72, 73]. Similarly, whether the use and application of an intersectional lens to research warrants an explicit commitment to social justice (the term justice is suggested to be interchangeable with equity [71]) has also been debated in the literature [19, 74, 75]. For example, Rice, Harrison et al. maintain that “intersectionality orients to social justice, so research utilizing intersectional analysis must commit to justice in its processes and knowledge production” [75]. By contrast, Collins claims that not all forms of intersectionality scholarship necessarily require an explicit commitment to social justice; however, a focus on social justice may offer a valuable unifying framework for intersectional inquiry [74]. Intersectionality scholars have also observed that as the concept of intersectionality has become more mainstream, its applications have increasingly drifted from intersectionality’s roots in Black feminist scholarship and social justice. Critiquing this shift, they use the terms “whitening” and “flattening” to refer to approaches to intersectionality that omit considerations of race and lack a focus on social justice and power [76, 77]. In our view, intersectionality research that de-emphasizes or disregards social justice limits its ability to contribute to social change. Researchers from all epistemological and ontological backgrounds must reflect on their own social positions and the factors which determine what is being researched and how – i.e. the contingency of (scientific) knowledge on power. Furthermore, epidemiological studies that seek to identify determinants of health/health inequities in the context of climate change should be informed by conceptual frameworks such as the CSDH framework for action on the social determinants of health and/or theories such as intersectionality. As Bowleg argued, “[p]ublic health’s commitment to social justice makes it a natural fit with intersectionality’s focus on multiple historically oppressed populations” [78]. Authors should engage in normative discussion of their results in reference to such theories and frameworks which in turn warrants interdisciplinary collaboration, e.g. with ethicists and social science scholars. This might help to clarify evitability of inequalities and hence identify responsible actors as well as effective interventions.

### Considerations for future research

#### Application of an intersectional lens to public health research

Consideration of determinants of inequalities is required in all public health research. Using logic models could help to comprehensively conceptualize policy effects and equity impacts thereof [79]. Furthermore, in taking an intersectional approach, attention should be directed towards identifying the (implicit) normative models that lead to structurally determined inequalities in the researched context. This calls for the use of mixed-methods approaches that seek to not only quantify heat-related health inequalities but also establish why they arise (beyond looking at associations between different variables and health outcomes) and whether and to what extent they are policy-amenable. Further development of (quantitative) methods to assess health burdens in groups affected differentially by systems of power and oppression, such as racism, sexism, and classism, and critical reflection on epistemological underpinnings is desirable [37, 80, 81]. Such endeavours should themselves be spearheaded by diverse groups of scientists who can harness the knowledge already available beyond the English, peer-reviewed literature. The development of guidance for carrying out literature reviews with an intersectional lens would be a valuable contribution.

#### Methodological considerations for research on the health impacts of heat

Additional literature reviews summarising evidence from outside the WHO European region could be carried out and specifically assess the transferability of findings to various local contexts, e.g. by developing typologies of likely occurring health inequalities by context (e.g. climatic or in terms of governance of health and nursing care structures). Currently underinvestigated factors such as differential exposures through work and physical environments clearly require further investigation in terms of their effects on health outcomes, as well as their amenability through generic or tailored measures. At the level of data collection and data analysis, structural determinants of health ought to be considered, with particular attention to the operationalisation of gender versus sex as stratifiers.

### Strengths and limitations

Transparent and replicable review procedures were applied. A process of critically reflecting on our own positionality and our approach to intersectionality accompanied the research process.

We applied a limited operationalization of health, i.e. we did not include studies that used proxies for health such as loss of productivity as outcomes. We also excluded studies that assessed effects of heat in interaction with other exposures such as air pollution. Despite including studies from the whole WHO European region, for feasibility reasons, we limited our searches to studies published in English or German and ran the search strings in English only. For the same reasons, no grey literature was included, and no forward or backward citation searches were carried out.

We systematically searched two scientific databases and applied broad inclusion criteria at the title/abstract screening stage to include all potentially relevant studies. We deliberately chose Pubmed and Epistemonikos to ensure retrieval of (public) health-related research. Epistemonikos contains (public) health-related reviews only and is therefore highly useful to meet our study aim. Searching further scientific databases such as Scopus or CINAHL might have yielded more studies.

We chose to not pre-emptively define populations of interest to not limit findings to those groups we know or assumed to be vulnerable in the context of heat. This reflects our understanding that vulnerability is not a fixed characteristic of any population group but rather contingent on the hazard, the social position in societal structures and the adaptive capacity of the exposed.

We did not assess whether study authors implicitly applied an intersectional perspective to their work, e.g. by screening for wordings that might reflect this as we followed Bauer et al.’s assessment that “a lack of any definition […], non-citation of foundational authors […] or of any intersectionality methods papers” [37] points to a shallow or no engagement with intersectionality theory. However, this approach might have led to undue misinterpretation of authors’ intentions.

We did not carry out a risk of bias assessment as per established guidance for scoping reviews [24].

Relatively few studies conducted subgroup analyses or reported on sub-groups which could be due to small sample sizes and expected insignificant results for subgroups or because intersectionality was not used as a foundational paradigm.

## Conclusions

To our knowledge, this scoping review of reviews is the first attempt to carry out a literature review with an intersectional lens in the context of heat-related health inequalities in the WHO European Region. This overview of the available evidence can serve to develop working hypotheses by policymakers and practitioners regarding target groups for interventions. However, this evidence base is characterised by important limitations: it lacks analyses that address why health inequalities arise and whether they are amenable to (policy) intervention. Identifying inequalities as inequities based on an evidence-informed normative analysis could present an important aid to policymakers.

Beyond age and sex/gender, other determinants of heat-related health inequalities are assessed to a lesser extent in this literature, as are the intersections between diverse axes of inequality. This points to a need for more comprehensive data collection and innovative methodological assessment of such data to provide a more granular perspective that reflects modern understandings of health and determinants of health and health inequities.

Our findings underscore the need for more research grounded in intersectionality theory to reveal and address the disproportionate burdens faced by groups with intersecting social identities as well as for methodological guidance on intersectionality-informed reviews.

Understanding who is most affected and why is key so that those most affected by heat-related health effects can be effectively protected and differences in disease burden can be causally addressed.

## Supplementary Information

Below is the link to the electronic supplementary material.

Supplementary Material 1

## Data Availability

No datasets were generated or analysed during the current study.

## Footnotes

1 PROGRESS-Plus: **P**lace of residence, **R**ace/ethnicity/culture/language, **O**ccupation, **G**ender/sex, **R**eligion, **E**ducation, **S**ocioeconomic status, **S**ocial capital, **Plus** (personal characteristics associated with discrimination, features of relationships, time-dependent relationships).

## References

[1] Watts N, et al. The 2020 report of The Lancet Countdown on health and climate change: responding to converging crises. Lancet. 2021;397(10269):129–70. 33278353 10.1016/S0140-6736(20)32290-X PMC7616803

[2] Romanello M, et al. The 2022 report of The Lancet Countdown on health and climate change: health at the mercy of fossil fuels. Lancet. 2022;400(10363):1619–54. 36306815 10.1016/S0140-6736(22)01540-9 PMC7616806

[3] Intergovernmental Panel on Climate Change. Climate Change 2022 – Impacts, Adaptation and Vulnerability: Working Group II Contribution to the Sixth Assessment Report of the Intergovernmental Panel on Climate Change. Cambridge: Cambridge University Press; 2023.

[4] Lee H et al. IPCC, 2023: Climate change 2023: Synthesis report, summary for policymakers. Contribution of working groups i, II and III to the sixth assessment report of the intergovernmental panel on climate change [core writing team, h. Lee and j. Romero, editors]. IPCC, geneva, Switzerland. 2023.

[5] Guerreiro SB, et al. Future heat-waves, droughts and floods in 571 European cities. Environ Res Lett. 2018;13(3):034009.

[6] Agency EE. European Climate Risk Assessment (EEA Report 01/2024). Publications Office of the European Union Luxembourg; 2024.

[7] Blom I, Robin F. Understanding climate-related threats to health in the WHO European Region. WHO Regional Office for Europe: Copenhagen; 2025.

[8] Romanello M, et al. The 2025 report of the Lancet Countdown on health and climate change. The Lancet, 2025. 10.1016/S0140-6736(25)01919-1 41175887

[9] Ebi KL, et al. Hot weather and heat extremes: health risks. lancet. 2021;398(10301):698–708. 34419205 10.1016/S0140-6736(21)01208-3

[10] Paavola J. Health impacts of climate change and health and social inequalities in the UK. Environ Health. 2017;16(1):113. 29219089 10.1186/s12940-017-0328-z PMC5773866

[11] Solar O, Irwin A. A conceptual framework for action on the social determinants of health. WHO Document Production Services; 2010.

[12] Miani C, et al. Measurement of gender as a social determinant of health in epidemiology—A scoping review. PLoS ONE. 2021;16(11):e0259223. 34731177 10.1371/journal.pone.0259223 PMC8565751

[13] McCartney G, et al. Defining health and health inequalities. Public Health. 2019;172:22–30. 31154234 10.1016/j.puhe.2019.03.023 PMC6558275

[14] Whitehead M. The Concepts and Principles of Equity and Health. Int J Health Serv. 1992;22(3):429–45. 1644507 10.2190/986L-LHQ6-2VTE-YRRN

[15] World Health Organization (WHO). Health equity. 2025 [cited 2025 9 November]; Available from: https://www.who.int/health-topics/health-equity#tab=tab_1

[16] Vallgårda S. When are health inequalities a political problem? Eur J Public Health. 2006;16(6):615–6. 16601106 10.1093/eurpub/ckl047

[17] National Collaborating Centre for Determinants of Health. Let’s Talk: Intersectionality. 2022. [Cited 2025 9th November]; Available from: https://nccdh.ca/resources/entry/lets-talk-intersectionality/

[18] Crenshaw KW. Mapping the margins: Intersectionality, identity politics, and violence against women of color, in The public nature of private violence. Routledge; 2013. pp. 93–118.

[19] Kelly C, et al. Doing’or ‘using’intersectionality? Opportunities and challenges in incorporating intersectionality into knowledge translation theory and practice. Int J Equity Health. 2021;20(1):187. 34419053 10.1186/s12939-021-01509-z PMC8379861

[20] Collins PH, Bilge S. Intersectionality. Wiley; 2020.

[21] Diderichsen F, et al. Health Inequality - determinants and policies. Scand J Public Health. 2012;40(8suppl):12–105. 23147863 10.1177/1403494812457734

[22] Bolte G, et al. Climate change and health equity: A public health perspective on climate justice. J health Monit. 2023;8(Suppl 6):3. 38105794 10.25646/11772 PMC10722520

[23] Casey JA, et al. Methods in Public Health Environmental Justice Research: a Scoping Review from 2018 to 2021. Curr Environ Health Rep. 2023;10(3):312–36. 37581863 10.1007/s40572-023-00406-7 PMC10504232

[24] Arksey H, O’Malley L. Scoping studies: towards a methodological framework. Int J Soc Res Methodol. 2005;8(1):19–32.

[25] Peters MD et al. Chap. 11: Scoping reviews (2020 version), in JBI manual for evidence synthesis, E. Aromataris and Z. Munn, Editors. 2020.

[26] Leppmeier L, et al. Heat, health and inequalities - a rapid overview of systematic reviews and recent primary studies from an intersectional perspective. 2023. https://osf.io/ychfj

[27] Tricco AC, et al. PRISMA extension for scoping reviews (PRISMA-ScR): checklist and explanation. Ann Intern Med. 2018;169(7):467–73. 30178033 10.7326/M18-0850

[28] Diderichsen F, Hallqvist J. Social inequalities in health some methodological considerations for the study of social position and social context. Inequality in Health - A Swedish Perspective. Swedish Council for Social Research: Stockholm; 1998. pp. 25–39.

[29] O’Neill J, et al. Applying an equity lens to interventions: using PROGRESS ensures consideration of socially stratifying factors to illuminate inequities in health. J Clin Epidemiol. 2014;67(1):56–64. 24189091 10.1016/j.jclinepi.2013.08.005

[30] World Health Organisation. Countries. 2025 [cited 2025 17th February]; Available from: https://www.who.int/countries/

[31] Conti A, et al. Knowledge Gaps and Research Priorities on the Health Effects of Heatwaves: A Systematic Review of Reviews. Int J Environ Res Public Health. 2022;19(10):5887. 35627424 10.3390/ijerph19105887 PMC9140727

[32] Slesinski SC, et al. Social inequalities in exposure to heat stress and related adaptive capacity: A systematic review. Environmental Research Letters; 2025.

[33] The Endnote Team. 2013, Clarivate: Philadelphia, PA.

[34] Ouzzani M, et al. Rayyan—a web and mobile app for systematic reviews. Syst reviews. 2016;5:1–10. 10.1186/s13643-016-0384-4 PMC5139140 27919275

[35] Bowleg L. When Black + Lesbian + Woman ≠ Black Lesbian Woman: The Methodological Challenges of Qualitative and Quantitative Intersectionality Research. Sex Roles. 2008;59(5):312–25.

[36] Alvidrez J, et al. Intersectionality in public health research: A view from the National Institutes of Health. 2021, American Public Health Association. pp. 95–97. 10.2105/AJPH.2020.305986 PMC7750592 33326274

[37] Bauer GR, et al. Intersectionality in quantitative research: A systematic review of its emergence and applications of theory and methods. SSM-population health. 2021;14:100798. 33997247 10.1016/j.ssmph.2021.100798 PMC8095182

[38] Public Health Agency of Canada. How to integrate intersectionality theory in quantitative health equity analysis? A rapid review and checklist of promising practices. PHAC: Ottawa; 2022.

[39] Alentorn A, et al. Spatial and Ecological Factors Modulate the Incidence of Anti-NMDAR Encephalitis—A Systematic Review. Biomedicines. 2023;11(6):1525. 37371620 10.3390/biomedicines11061525 PMC10295747

[40] Fatima SH, et al. Extreme heat and occupational injuries in different climate zones: A systematic review and meta-analysis of epidemiological evidence. Environ Int. 2021;148:106384. 33472088 10.1016/j.envint.2021.106384

[41] Perry T, Obolski U, Peretz C. The association between high ambient temperature and mortality in the Mediterranean Basin: a systematic review and meta-analysis. Curr Environ health Rep. 2023;10(1):61–71. 36417094 10.1007/s40572-022-00386-0

[42] Witt C, et al. The effects of climate change on patients with chronic lung disease: a systematic literature review. Deutsches Ärzteblatt international. 2015;112(51–52):878. 26900154 10.3238/arztebl.2015.0878 PMC4736555

[43] Byun G, et al. Effects of ambient temperature on mental and neurological conditions in older adults: A systematic review and meta-analysis. Environ Int. 2024;194:109166. 39603080 10.1016/j.envint.2024.109166 PMC11675999

[44] Krishnakumar M, et al. Impact of exposure to extreme heat events during pregnancy on the incidence of congenital heart disease in offspring: a meta-analysis. BMJ Paediatrics Open. 2024;8(1):e002848. 39653516 10.1136/bmjpo-2024-002848 PMC11629000

[45] Tran HM et al. Corrigendum to ‘Extreme temperature increases the risk of COPD morbimortality: A systematic review and meta-analysis [Science of The Total Environment, Vol 958 [2025] 178087’. Science of The Total Environment, 2025: p. 178996. 10.1016/j.scitotenv.2025.178996 40087052

[46] Zhou Y, et al. Sex differences in the impact of extreme heat on cardiovascular disease outcomes: a systematic review and meta-analysis. Environ Health. 2025;24(1):20. 40221760 10.1186/s12940-025-01175-6 PMC11992858

[47] Gupta S et al. Electric fans for reducing adverse health impacts in heatwaves. Cochrane Database Syst Reviews, 2012(7). 10.1002/14651858.CD009888.pub2 PMC6457598 22786530

[48] Haghighi MM, et al. Impacts of high environmental temperatures on congenital anomalies: a systematic review. Int J Environ Res Public Health. 2021;18(9):4910. 34063033 10.3390/ijerph18094910 PMC8124753

[49] Hedlund C, Blomstedt Y, Schumann B. Association of climatic factors with infectious diseases in the Arctic and subarctic region–a systematic review. Global Health Action. 2014;7(1):24161. 24990685 10.3402/gha.v7.24161 PMC4079933

[50] Levi M, Kjellstrom T, Baldasseroni A. Impact of climate change on occupational health and productivity: a systematic literature review focusing on workplace heat. La Medicina del Lavoro. 2018;109(3):163. 29943748 10.23749/mdl.v109i3.6851 PMC7689800

[51] Weilnhammer V, et al. Extreme weather events in europe and their health consequences–A systematic review. Int J Hyg Environ Health. 2021;233:113688. 33530011 10.1016/j.ijheh.2021.113688

[52] Dickinson N, et al. Extreme weather events in the UK and resulting public health outcomes. Int J Public Health. 2025;70:1607904. 40510033 10.3389/ijph.2025.1607904 PMC12158791

[53] Pantavou K, et al. Thermal indices for evaluating the impact of thermal conditions on human health: A systematic review. Eur J Pub Health. 2024;34(Supplement3):ckae144.

[54] Cicci KR, et al. High temperatures and cardiovascular-related morbidity: a scoping review. Int J Environ Res Public Health. 2022;19(18):11243. 36141512 10.3390/ijerph191811243 PMC9517671

[55] Massazza A, Ardino V, Fioravanzo RE. Climate change, trauma and mental health in Italy: a scoping review. Eur J psychotraumatology. 2022;13(1):2046374. 10.1080/20008198.2022.2046374 PMC9009940 35432785

[56] Paterson SK, Godsmark CN. Heat-health vulnerability in temperate climates: lessons and response options from Ireland. Globalization health. 2020;16:1–17. 32228631 10.1186/s12992-020-00554-7 PMC7106697

[57] Edwards JR, et al. Residential indoor temperatures and health: A scoping review of observational studies. Sci Total Environ. 2025;979:179377. 40286610 10.1016/j.scitotenv.2025.179377 PMC13090820

[58] Meherali S, et al. Impact of climate change on maternal health outcomes: An evidence gap map review. PLOS Global Public Health. 2024;4(8):e0003540. 39159145 10.1371/journal.pgph.0003540 PMC11332935

[59] Wu WJ, et al. Scoping review of the characteristics and outcomes of adults presenting to the emergency department during heatwaves. Emerg Med Australasia. 2023;35(6):903–20. 10.1111/1742-6723.14317 37788821

[60] Gebhardt N, et al. Scoping review of climate change and mental health in Germany - Direct and indirect impacts, vulnerable groups, resilience factors. J Health Monit. 2023;8(Suppl 4):122–49. 37799536 10.25646/11656 PMC10548489

[61] Anderson M, et al. Defining indoor heat thresholds for health in the UK. Perspect public health. 2013;133(3):158–64. 22833542 10.1177/1757913912453411

[62] Arbuthnott KG, Hajat S. The health effects of hotter summers and heat waves in the population of the United Kingdom: a review of the evidence. Environ Health. 2017;16:1–13. 29219088 10.1186/s12940-017-0322-5 PMC5773858

[63] Bittner MI. [Effects of heat waves on mortality in Germany]. Gesundheitswesen. 2014;76(8–9):508–12. 24163214 10.1055/s-0033-1355404

[64] Green H, et al. Impact of heat on mortality and morbidity in low and middle income countries: a review of the epidemiological evidence and considerations for future research. Environ Res. 2019;171:80–91. 30660921 10.1016/j.envres.2019.01.010

[65] Martiello MA, Giacchi MV. High temperatures and health outcomes: a review of the literature. Scand J Public Health. 2010;38(8):826–37. 20688791 10.1177/1403494810377685

[66] van Steen Y, et al. Sex differences in mortality after heat waves: are elderly women at higher risk? Int Arch Occup Environ Health. 2019;92:37–48. 30293089 10.1007/s00420-018-1360-1

[67] Mena E, Bolte G, on behalf of the ADVANCE GENDER Study Group. Intersectionality-based quantitative health research and sex/gender sensitivity: a scoping review. Int J Equity Health. 2019;18(1):199. 31864366 10.1186/s12939-019-1098-8 PMC6925460

[68] Quesada J, Hart LK, Bourgois P. Structural vulnerability and health: Latino migrant laborers in the United States. Med Anthropol. 2011;30(4):339–62. 21777121 10.1080/01459740.2011.576725 PMC3146033

[69] Slesinski CS et al. Social inequalities in exposure to heat stress and related adaptive capacity: a systematic review. Environ Res Lett, 2025. 20(3).

[70] Amorim-Maia AT et al. Intersectional climate justice: A conceptual pathway for bridging adaptation planning, transformative action, and social equity. Urban Clim, 2022. 41(101053).

[71] Braveman P. Health Inequalities, Disparities, Equity: What’s in a Name? 2025, American Public Health Association. pp. 996–1002. 10.2105/AJPH.2025.308062 PMC12160630 40499108

[72] Bhopal A. Are you a researcher or an activist?’: Navigating tensions in climate change and health research. J Clim Change Health. 2023;14:100267.

[73] Gardner CJ, et al. From publications to public actions: the role of universities in facilitating academic advocacy and activism in the climate and ecological emergency. Front Sustain. 2021;2:679019.

[74] Collins PH. Intersectionality as critical social theory. Duke University Press; 2019.

[75] Rice C, Harrison E, Friedman M. Doing justice to intersectionality in research. Cult Studies↔ Crit Methodologies. 2019;19(6):409–20.

[76] Bilge S. Whitening intersectionality: Evanescence of race in intersectionality scholarship. Racism Sociol. 2014;5:175–206.

[77] Bowleg L. Evolving Intersectionality Within Public Health: From Analysis to Action. Am J Public Health. 2021;111(1):88–90. 33326269 10.2105/AJPH.2020.306031 PMC7750585

[78] Bowleg L. The Problem With the Phrase Women and Minorities: Intersectionality—an Important Theoretical Framework for Public Health. Am J Public Health. 2012;102(7):1267–73. 22594719 10.2105/AJPH.2012.300750 PMC3477987

[79] Sell K, Rabbani S, Burns J. How is health equity considered in policy evaluations employing quasi-experimental methods? A scoping review and content analysis. Eur J Pub Health. 2024;35(1):42–51. 10.1093/eurpub/ckae188 PMC11832135 39602551

[80] Martinez Dy A, Martin L, Marlow S. Developing a critical realist positional approach to intersectionality. J Crit realism. 2014;13(5):447–66.

[81] Fehrenbacher AE, Patel D. Translating the theory of intersectionality into quantitative and mixed methods for empirical gender transformative research on health. Cult Health Sex. 2020;22(sup1):145–60. 31661661 10.1080/13691058.2019.1671494 PMC7188600

