# Supplementary material for: Heat, health and inequalities in the WHO European region – a scoping review with an intersectional lens
Source: Int J Equity Health. 2026 Feb 23;25:60. doi: 10.1186/s12939-026-02787-1 (PMC12955224; doi:10.1186/s12939-026-02787-1)
Supplement: Supplementary file 1 — Supplementary Material 1 [file 12939_2026_2787_MOESM1_ESM.docx]

Appendices

Appendix 1: Search String Reviews (PubMed)

("heat wave*"[All Fields] OR "heatwave*"[All Fields] OR "extreme heat*"[All Fields] OR "extreme heat*"[MeSH Terms] OR "hot weather*"[All Fields] OR "hot temperature*"[All Fields] OR "high temperature*"[All Fields] OR "extreme temperature*"[All Fields] OR "warm temperature*"[All Fields] OR "extreme weather*"[All Fields] OR "temperature-mortalit*"[All Fields] OR "temperature-morbidit*"[All Fields] OR "temperature-related*"[All Fields] OR heat*[All Fields] OR “non-optimal temperature*”[All Fields] OR “hot day*”[All Fields] OR “hot day”[All Fields] OR “heat exposure*”[All Fields] OR “heat risk*”[All Fields] OR “apparent temperature*”[All Fields] OR “heat stress*”[All Fields] OR “heat strain*”[All Fields] OR “equivalent temperature*”[All Fields] OR “ambient temperature*”[All Fields] OR “wet bulb globe temperature*”[All Fields] OR WBGT*[All Fields] OR “urban heat island*”[All Fields] OR “heat index*”[All Fields] OR “temperature humidity index*”[All Fields] OR “comfort index*”[All Fields] OR “discomfort index*”[All Fields] OR “universal thermal climate index*”[All Fields] OR UTCI*[All Fields] OR humidex*[All Fields])

AND ("health"[MeSH Terms] OR "health*"[All Fields] OR “morbidit*”[All Fields] OR “mortalit*”[All Fields] OR “illness*”[All Fields] OR “disease*”[All Fields])

AND (meta-analysis[Filter] OR review[Filter] OR systematicreview[Filter])

AND (Albania[Title/Abstract] OR Andorra[Title/Abstract] OR Armenia[Title/Abstract] OR Austria[Title/Abstract] OR Azerbaijan[Title/Abstract] OR Republic of Belarus[Title/Abstract] OR Belgium[Title/Abstract] OR Bosnia and Herzegovina[Title/Abstract] OR Bulgaria[Title/Abstract] OR Croatia[Title/Abstract] OR Cyprus[Title/Abstract] OR Czech Republic[Title/Abstract] OR Denmark[Title/Abstract] OR Estonia[Title/Abstract] OR Finland[Title/Abstract] OR France[Title/Abstract] OR Georgia[Title/Abstract] OR Germany[Title/Abstract] OR Greece[Title/Abstract] OR Hungary[Title/Abstract] OR Iceland[Title/Abstract] OR Ireland[Title/Abstract] OR Israel[Title/Abstract] OR Italy[Title/Abstract] OR Kazakhstan[Title/Abstract] OR Kyrgyzstan[Title/Abstract] OR Latvia[Title/Abstract] OR Lithuania[Title/Abstract] OR Luxembourg[Title/Abstract] OR Malta[Title/Abstract] OR Monaco[Title/Abstract] OR Montenegro[Title/Abstract] OR Netherlands[Title/Abstract] OR Republic of North Macedonia[Title/Abstract] OR Norway[Title/Abstract] OR Poland[Title/Abstract] OR Portugal[Title/Abstract] OR Moldova[Title/Abstract] OR Romania[Title/Abstract] OR Russia[Title/Abstract] OR San Marino[Title/Abstract] OR Serbia[Title/Abstract] OR Slovakia[Title/Abstract] OR Slovenia[Title/Abstract] OR Spain[Title/Abstract] OR Sweden[Title/Abstract] OR Switzerland[Title/Abstract] OR Tajikistan[Title/Abstract] OR Turkey[Title/Abstract] OR Turkmenistan[Title/Abstract] OR Ukraine[Title/Abstract] OR United Kingdom[Title/Abstract] OR Uzbekistan[Title/Abstract] OR Europe*[Title/Abstract] OR "Albania"[MeSH] OR "Andorra"[MeSH] OR "Armenia"[MeSH] OR "Austria"[MeSH] OR "Azerbaijan"[MeSH] OR "Republic of Belarus"[MeSH] OR "Belgium"[MeSH] OR "Bosnia and Herzegovina"[MeSH] OR "Bulgaria"[MeSH] OR "Croatia"[MeSH] OR "Cyprus"[MeSH] OR "Czech Republic"[MeSH] OR "Denmark"[MeSH] OR "Estonia"[MeSH] OR "Finland"[MeSH] OR "France"[MeSH] OR "Georgia"[MeSH] OR "Germany"[MeSH] OR "Greece"[MeSH] OR "Hungary"[MeSH] OR "Iceland"[MeSH] OR "Ireland"[MeSH] OR "Israel"[MeSH] OR "Italy"[MeSH] OR "Kazakhstan"[MeSH] OR "Kyrgyzstan"[MeSH] OR "Latvia"[MeSH] OR "Lithuania"[MeSH] OR "Luxembourg"[MeSH] OR "Malta"[MeSH] OR "Monaco"[MeSH] OR "Montenegro"[MeSH] OR "Netherlands"[MeSH] OR "Republic of North Macedonia"[MeSH] OR "Norway"[MeSH] OR "Poland"[MeSH] OR "Portugal"[MeSH] OR "Moldova"[MeSH] OR "Romania"[MeSH] OR "Russia"[MeSH] OR "San Marino"[MeSH] OR "Serbia"[MeSH] OR "Slovakia"[MeSH] OR "Slovenia"[MeSH] OR "Spain"[MeSH] OR "Sweden"[MeSH] OR "Switzerland"[MeSH] OR "Tajikistan"[MeSH] OR "Turkey"[MeSH] OR "Turkmenistan"[MeSH] OR "Ukraine"[MeSH] OR "United Kingdom"[MeSH] OR "Uzbekistan"[MeSH] OR "Europe”[MeSH])

AND ((English[Language]) OR (German[Language]))

Appendix 2: Search String Reviews (Epistemonikos)

("heat wave*" OR "heatwave*" OR "extreme heat*" OR "hot weather*" OR "hot temperature*" OR "high temperature*" OR "extreme temperature*" OR "warm temperature*" OR "extreme weather*" OR "temperature-mortalit*" OR "temperature-morbidit*" OR "temperature-related*" OR heat* OR "non‐optimal temperature*" OR "hot days*" OR "hot day*" OR "heat exposure*" OR "heat risk*" OR "apparent temperature*" OR "heat stress*" OR "heat strain*" OR "equivalent temperature*" OR "ambient temperature*" OR "wet bulb globe temperature*" OR (WBGT*) OR "urban heat island*" OR "heat index*" OR "temperature humidity index*" OR "comfort index*" OR "discomfort index*" OR "universal thermal climate index*" OR (UTCI*) OR (humidex*)) AND ("health*" OR "morbidit*" OR "mortalit*" OR "illness*" OR "disease*") AND (Albania OR Andorra OR Armenia OR Austria OR Azerbaijan OR Republic of Belarus OR Belgium OR (Bosnia AND Herzegovina) OR Bulgaria OR Croatia OR Cyprus OR Czech Republic OR Denmark OR Estonia OR Finland OR France OR Georgia OR Germany OR Greece OR Hungary OR Iceland OR Ireland OR Israel OR Italy OR Kazakhstan OR Kyrgyzstan OR Latvia OR Lithuania OR Luxembourg OR Malta OR Monaco OR Montenegro OR Netherlands OR Republic of North Macedonia OR Norway OR Poland OR Portugal OR Moldova OR Romania OR Russia OR San Marino OR Serbia OR Slovakia OR Slovenia OR Spain OR Sweden OR Switzerland OR Tajikistan OR Turkey OR Turkmenistan OR Ukraine OR United Kingdom OR Uzbekistan OR Europe OR "Albania" OR "Andorra" OR "Armenia" OR "Austria" OR "Azerbaijan" OR "Republic of Belarus" OR "Belgium" OR "Bosnia AND Herzegovina" OR "Bulgaria" OR "Croatia" OR "Cyprus" OR "Czech Republic" OR "Denmark" OR "Estonia" OR "Finland" OR "France" OR "Georgia" OR "Germany" OR "Greece" OR "Hungary" OR "Iceland" OR "Ireland" OR "Israel" OR "Italy" OR "Kazakhstan" OR "Kyrgyzstan" OR "Latvia" OR "Lithuania" OR "Luxembourg" OR "Malta" OR "Monaco" OR "Montenegro" OR "Netherlands" OR "Republic of North Macedonia" OR "Norway" OR "Poland" OR "Portugal" OR "Moldova" OR "Romania" OR "Russia" OR "San Marino" OR "Serbia" OR "Slovakia" OR "Slovenia" OR "Spain" OR "Sweden" OR "Switzerland" OR "Tajikistan" OR "Turkey" OR "Turkmenistan" OR "Ukraine" OR "United Kingdom" OR "Uzbekistan" OR "Europe")

Appendix 3: Categories for data extraction

| **Study basics** | **Authors** |
| --- | --- |
|  | **Title** |
|  | **Publication date** |
|  | **Study design as reported by authors** |
|  | **Journal** |
| **Study characteristics** | **Study aim(s), research question or objectives** |
|  | **Number of studies included** |
|  | **Time frame [in years] of the publication years of studies included** |
|  | **Assessment of quality [y/n] and if yes, how?** |
|  | **Inclusion of a meta-analysis or any other synthesis** |
|  | **General policy recommendations** |
|  | **Comments** |
| **Population and setting** | **Geographic focus** |
|  | **Setting** |
|  | **Population** |
|  | **Population size** |
|  | **Other context** |
|  | **Comments** |
| **Variables** | **Health as mediator, outcome, exposure?** |
|  | **Operationalization of health** |
|  | **Inequality/inequity as mediator, outcome, exposure?** |
|  | **Operationalization of inequality/inequity** |
|  | **Heat as mediator, exposure, outcome?** |
|  | **Operationalization of heat** |
|  | **Comments** |
| **Results** | **General results** |
|  | **Results per subgroup** |
|  | **Comments** |
| **Health inequalities: stratification by sub-population** | **Sex/gender** |
|  | **Age** |
|  | **Marital status/social network** |
|  | **Ethnicity** |
|  | **Education** |
|  | **Income** |
|  | **Work*** |
|  | **Area of living / housing conditions** |
|  | **Ecological parameters** |
|  | **Health status** |
|  | **SES** |
|  | **Other** |
|  | **Comments** |
| **Health equity concepts** | **Reflexivity statement by authors [y/n]** |
|  | **Inequalities considered as inequities [y/n]** |
|  | **How are inequalities discussed?** |
| **Data extraction reflection** | **Mechanisms that create inequalities/inquities as applied by the authors in methods and analysis** |
|  | **Mechanisms** |
|  | **Explicit use of intersectional lens/justice approach to research [y/n]** |
|  | **Which methods were used to operationalise intersectional lens** |
|  | **Solutions/approaches to address inequities/inequalities + relevant actor mentioned** |
|  | **Comments** |

Appendix 4: Overview of characteristics of reviews

| **Study basics** | | | | **Study characteristics** | | | **Population and Setting** | |
| --- | --- | --- | --- | --- | --- | --- | --- | --- |
| First author and year | Title | | | Study aim(s); Research question or objectives | Number of studies included | Assessment of quality and if yes how? (e.g. risk of bias tool) | Geographic focus (e.g. country/ countries) | Population(s) of interest |
| Systematic Review with meta-analysis | | | | | | | | |
| Alentorn et al. (2023) | Spatial and ecological factors modulate the incidence of Anti-NMDAR Encephalitis - A systematic review | | | To assess the incidence of anti-NMDAR encephalitis in different countries, searching for elements suggesting an influence from geographic, climatic, and environmental factors | 68 | nr | Worldwide with special focus on Greece and France; inclusion of studies from 30 different countries | General population |
| Byun et al. (2024)^ⴕ^ | Effects of ambient temperature on mental and neurological conditions in  older adults: A systematic review and meta-analysis | | | This systematic review and meta-analysis study aims to summarize  the epidemiological evidence of the effects of ambient temperature,  including both heat and cold exposures, on mental and neurological  conditions in older adults | 76 | Risk of bias (RoB) assessment tool developed by the World Health Organization | Global | Individuals aged 60 years and older, with no restrictions based on gender,  race, or ethnicity |
| Fatima et al. (2021) | Extreme heat and occupational injuries in different climate zones: A systematic review and meta-analysis of epidemiological evidence | | | To summarize the existing epidemiological evidence on the impact of extreme heat (hot temperatures and heatwaves) on occupational injuries in different climate zones and to assess the individual risk factors associated with workers and workplaces that contribute to heat-associated occupational injuries risks | 24; 22 for meta-analysis | Navigation Guide framework | Worldwide | Workers |
| Krishnakumar et al. (2024)^ⴕ^ | Impact of exposure to extreme heat events during pregnancy on the incidence of congenital heart disease in offspring: a meta-analysis | | | The primary objective of this meta-analysis is to quantify the extent of the association between maternal exposure to EHE during pregnancy and the likelihood of CHD in the foetus. | 8 | Newcastle Ottawa scale (NOS) | Global | Weeks 2–8 of the gestation period |
| Perry et al. (2023) | The association between high ambient temperature and mortality in the Mediterranean Basin: A systematic review and meta‑analysis | | | To quantify the risk of mortality associated with exposure to high ambient temperature in the Mediterranean basin in the general population and in vulnerable sub-populations | 16 | nr | Mediterranean basin; inclusion of studies from Europe (Cyprus, Greece, Italy, Portugal, Slovenia, and Spain), Asia (Israel, Lebanon, and Turkey) and North Africa (Tunisia) | General population and vulnerable sub-populations |
| Tran et al. (2025)^ⴕ^ | Corrigendum to ‘Extreme temperature increases the risk of COPD morbimortality: A systematic review and meta-analysis [Science of The Total Environment, Vol 958 [2025] 178087’ | | | This study aims to fill this gap by conducting a comprehensive systematic review and meta-analysis to assess the influence of extreme temperatures on COPD morbimortality risk globally. We also  aim to identify vulnerable subpopulations susceptible to these temperature extremes by performing subgroup analyses based on age, sex, and regions | 25 | Yes, but without a published concept | Global | COPD patients |
| Witt et al. (2015) | The effects of climate change on patients with chronic lung disease | | | To assess effects of heat waves on the morbidity and mortality of persons with chronic lung disease | 33 | yes, but without a published concept | Worldwide; inclusion of studies from Europe, Asia, Australia and America | Persons with chronic lung disease |
| Zhou et al. (2025)^ⴕ^ | Sex differences in the impact of extreme heat on cardiovascular disease outcomes: a systematic review and meta-analysis | | | This systematic review and meta-analysis aim to examine the sex-specific association between extreme heat exposure and cardiovascular disease outcomes. The focus of the analysis is synthesizing epidemiological evidence from the past decade in males and females and quantifying sex-specific relative risks of CVD responses to extreme heat exposure through meta-analysis | 79 | Navigation Guide criteria | Global | General population |
| Systematic Review without meta-analysis | | | | | | | | |
| Dickinson et al. (2025)^ⴕ^ | | Extreme Weather Events in the UK and  Resulting Public Health Outcomes | | To investigate the impacts of EWEs on the UK population, public  health and wellbeing outcomes and evaluate through a health-economic lens | 48 | JBI critical appraisal tools | United Kingdom | Population affected by extreme weather events |
| Gupta et al. (2021) | | Electric fans for reducing adverse health impacts in heatwaves (Review) | | To determine whether the use of electric fans contributes to, or impedes, heat loss at high ambient temperatures during a heatwave, and to contribute to the evidence base for the public health impacts of heatwaves | 0 | Yes, if there were studies included | Worldwide | General population |
| Haghighi et al. (2021) | | Impacts of high environmental temperatures on congenital anomalies: A systematic review | | To review evidence of associations between heat exposure (ambient or environmental) and the incidence of congenital anomalies | 13 | No, but highlighted particular concerns around study quality as relevant | Worldwide; included studies from the United States, Asia and the Middle East, Canada and Europe | Foetuses and embryos |
| Hedlund et al. (2014) | | Association of climatic factors with infectious diseases in the Arctic and Subarctic region – A systematic review | | To investigate the evidence for an association between climatic factors and infectious diseases, and to identify the most climate-sensitive diseases and vulnerable populations in the Arctic and subarctic region | 29 | SIGN methodology checklist | Arctic and subarctic region (Canada, Greenland, Iceland, Norway, Sweden, Finland, Alaska and the northern parts of Russia); included studies from Canada, Alaska, Sweden, Finland, Norway and Russia | General population |
| Levi et al. (2018) | | Impact of climate change on occupational health and productivity: A systematic literature review focusing on workplace heat | | To summarize the epidemiological evidence of the effects of climate change, with a special focus on high temperatures and heat waves, on workers’ health and productivity, in order to better inform health policies in the EU and beyond | 36 | nr | Worldwide; included studies from North America, Asia, Australia/New Zealand, Central America and Europe | Workers |
| Pantavou et al. (2025)^ⴕ^ | | Thermal indices for evaluating the impact of thermal conditions on human health: a systematic review | | The aim of this paper is to summarize the existing evidence on the statistical relationship, (i.e., association) between thermal indices and human health. This will help identify the most commonly used indices, thereby contributing to the standardization of information and facilitating easier comparison of results. Moreover, this review aims to popularize the use of thermal indices among medical scientists, public health professionals, epidemiologists, and policymakers. It will also examine which diseases have been studied in relation to thermal indices, aiming to consolidate this information and highlight gaps in the application of thermal indices in medical and public health research | 310 | United States National Institutes of Health (NIH) Quality Assessment Tool for Observational Cohort and Cross-Sectional Studies | Global | General population |
| Weilnhammer et al. (2021) | | Extreme weather events in Europe and their health consequences – A systematic review | | To assess the current literature about the association between these extreme weather events and their impact on the health of the European population | 35 | NICE quality appraisal checklist | Europe; included studies from Czech Republic, Finland, France, Germany, Greece, Hungary, Ireland, Italy, Netherlands, Portugal, Russia, Spain, Sweden and UK | General population |
| Scoping Review | | | | | | | | |
| Cicci et al. (2022) | | | High temperatures and cardiovascular-related morbidity: A scoping review | To synthesize studies assessing the relationships between high temperatures and cardiovascular disease (CVD)-related hospital encounters (i.e., emergency department (ED) visits or hospitalizations) in urban Canada and other comparable populations, and to identify areas for future research | 22 | nr | North America and Europe | General population |
| Edwards et al. (2025)^ⴕ^ | | | Residential indoor temperatures and health: A scoping review of observational studies | The objective of this review was to identify peer-reviewed studies of associations of warm indoor temperatures with mortality/morbidity outcomes or thermal comfort | 29 | nr | Global | General population |
| Gebhardt et al. (2023) ^ⴕ^ | | | Scoping review of climate change and mental health in Germany - Direct and indirect impacts, vulnerable groups, resilience factors | The aim was to develop a comprehensive overview of the state of research in order to derive recommendations for mitigating the negative consequences of climate change on the mental health of the German population. | 10 | nr | Germany | General population |
| Massazza et al. (2022) | | | Climate change, trauma and mental health in Italy: A scoping review | To provide a review of the original research literature on the relationship between climate change stressors and mental health in Italy, with a particular focus on trauma and PTSD and to encourage more research and policy action on the intersection between climate change and mental health | 21 | NIH Quality Assessment Tool for Case–Control Studies and the NIH Quality Assessment Tool for Observational Cohort and Cross-Sectional Studies | Italy | General population |
| Meherali et al. (2024)^ⴕ^ | | | Impact of climate change on maternal health outcomes: An evidence gap map review | This review aims to bridge this gap by systematically mapping the global evidence available on the impact of climate change on maternal well-being by generating the evidence gap map (EGM). | 133 | Assessment of Multiple Systematic Reviews 2 (AMSTAR) for systematic reviews (RCTs and non-RCTs), the Mixed Methods Appraisal Tool (MMAT) for primary studies (qualitative and quantitative), and a Qualitative Meta-Review Quality Assessment Tool for the qualitative synthesis. | Global | Women aged 15–49 |
| Paterson et al. (2020) | | | Heat-health vulnerability in temperate climates: lessons and response options from Ireland | To highlight the anticipated heat-health challenges in Ireland, and other temperate regions, by analyzing vulnerable groups and systems, and to identify a range of potential interventions that can begin to tackle this growing threat | 15 | nr | Ireland; included studies from other temperate climates | Populations vulnerable to heat (older people; chronically ill; infants, pregnant women and children; outdoor workers; socio-economic status and urban dwellers; food systems and the health sector) |
| Wu et al. (2023)^ⴕ^ | | | Scoping review of the characteristics  and outcomes of adults presenting to the emergency  department during heatwaves | The aim of this review is to identify, evaluate and summarise current literature regarding the main characteristics and outcomes of adult  patients attending EDs during  heatwaves, whereas comparing different heatwave definitions. Specifically,  we sought to undertake a subgroup analysis of at-risk populations, that  were anticipated to be more susceptible during heatwaves. | 31 | Mixed Methods Appraisal  Tool (MMAT) | Global | Patients admitted to ED |
| Narrative Review / Literature search | | | | | | | | |
| Anderson et al. (2013) | | | Defining indoor heat thresholds for health in the UK | To outline the need for indoor heat thresholds and to establish if they can be identified | 96 | nr | United Kingdom; included studies worldwide | General population |
| Arbuthnott et al. (2017) | | | The health effects of hotter summers and heat waves in the population of the United Kingdom: A review of the evidence | To bring together evidence from epidemiological studies and health impact assessments to provide an overview of what is known about effects of heat on population-level health in the UK | 21 + 7 | nr | United Kingdom | General population |
| Bittner (2014) | | | Effects of heat waves on mortality in Germany [Auswirkungen von Hitzewellen auf die Mortalität in Deutschland] | To summarize and critically evaluate the results available for Germany on the subject of heatwave-associated mortality | 12 | nr | Germany | General population |
| Green et al. (2019) | | | Impact of heat on mortality and morbidity in low and middle income countries: A review of the epidemiological evidence and considerations for future research | To review the existing evidence in the literature on the impact of heat on human health in low and middle income countries | 146, 7 from Europe | nr | Worldwide (low- and middle-income countries); included studies from Asia, Sub-Saharan Africa, the Middle East and North Africa, Latin America and Europe | General population |
| Martiello et al. (2010) | | | High temperatures and health outcomes: A review of the literature | To review the epidemiological evidence of the health impact of high temperatures on the population, as well as the associated risk and protective factors | 113 | nr | Worldwide | General population |
| van Steen et al. (2019) | | | Sex differences in mortality after heat waves: Are elderly women at higher risk? | To review the presence of sex-specific results in studies performed on mortality in elderly (> 65 years old) after heat waves in Europe | 13 | nr | European countries | Population >=65 |

^ⴕ^ retrieved through updated search 9/2025

Appendix 5: Overview of characteristics of primary studies

|  | **Study basics** | | **Study characteristics** | | | **Population and Setting** | |
| --- | --- | --- | --- | --- | --- | --- | --- |
| Study ID | First author and year | Title | Study aim(s) | Time of data collection | Data sources | Geographic focus (e.g. country/ countries) | Population(s) of interest |
| Ecological design | | | | | | | |
| Astone_2022 | Astone et al. (2022) | Climate change and health: Consequences of high temperatures among vulnerable groups in Finland | To examine the causal effects of high temperatures on health care use and mortality in Finland, by establishing the overall effect on the whole population and identifying risk groups | 1998-2017; summer (may - september) | Population: Finnish Institute for Health and Welfare; Statistics Finland  Meteorological: Finnish Meteorological Institute | Finland | General population |
| Ballester_2023 | Ballester et al. (2023) | Heat-related mortality in Europe during the summer of 2022 | To quantify heat-related sex- and age-specific mortality burden during the summer of 2022, the hottest season on record in Europe | between 30 May and 4 September 2022 (weeks 22–35). | Population: Eurostat 46. -> Missing data: contacting the corresponding national agencies for statistics. | Europe | General population |
| Chitu_2023 | Chitu et al. (2023) | Large sex differences in vulnerability to circulatory-system disease under current and future climate in Bucharest and its rural surroundings | To assess the observed and future impact of temperature on CSD-driven daily mortality on vulnerable groups including less-analyzed ones such as women in the urban area of Bucharest (Romania) and nearby rural regions | 1999-2019 | Meteorological: weather station Bucuresti Filaret (urban); five stations about 50 km from Bucharest ((Oltenita, Videle, Alexandria, Urziceni and Titu) -> rural Climate projections: EURO-CORDEX Population: National Institute for Public Health (INSP) (mortality) | Rumania (Bucharest and its rural surroundings up to 50 km away) | General population |
| Choi_2022 | Choi et al. (2022) | Effect modification of greenness on the association between heat and mortality: A multi-city multi-country study | To examinate the heat-mortality relationship among different greenspace levels in a global setting | 2000-2018 | Population: Multi-Country Multi City (MCC) Collaborative Research Network (MCC) study Greeness: Enhanced Vegetation Index (EVI) and Normalized Difference Vegetation Index (NDVI) from the Moderate Resolution Imaging Spectroradiometer product MOD13Q1 | Worldwide; 452 cities from 24 countries | General population |
| Coma_2023 | Coma et al. (2023) | Mortality in Catalonia during the summer of 2022 and its relation with high temperatures and COVID-19 cases | To analyse the possible association between the increased mortality in Catalonia during the summer months of 2021 and 2022 and either higher than average temperatures or the COVID-19 wave | Meteorological summer months of the years 2021 and 2022, from 1st June to 31st August. | Meteorological: network of automatic weather stations; Catalan Open Data website Population: official register of insured persons (mortality); official Catalan infections surveillance system (influenza-like illness, COVID-19 cases and deaths) | Spain (Catalonia) | General population |
| Conte_2022 | Conte Keivabu (2022) | Extreme temperature and mortality by educational attainment in Spain, 2012–2018 | To provide new evidence on how socioeconomic status measured using the educational attainment of the deceased individual stratifies the relationship between temperature and mortality for individuals aged above 65 | January 2012-December 2018 | Population: Spanish National Statistics (Instituto Nacional de Estadistica—INE) (mortality); Spanish Labor Force Survey (Encuesta de Poblacion Activa—EPA) (sociodemographic characteristics, educational attainment and the causes of death) Meteorological: E-OBS  Pollution: Copernicus Atmosphere Data Store (CAMS) | Spain | Population aged above 65 |
| de Schrijver_2023 | de Schrijver et al. (2023) | Exploring vulnerability to heat and cold across urban and rural populations in Switzerland | To assess how heat- and cold-mortality risks change across urban, peri-urban and rural areas in Switzerland and to identify and compare the factors associated with increased vulnerability within and between different area typologies | 1.1.1990-31.12.2017 | Population: Swiss Federal Bureau of Statics (BFS) (morality).  Meteorological: gridded climate dataset (MeteoSwiss-grid-product) developed by MeteoSwiss | Switzerland | General population |
| de Visser_2023 | de Visser et al. (2023) | Geographic and socioeconomic differences in heat-related mortality among the Dutch population: A time series analysis | To examine modification in heat-related mortality in the Netherlands by sociodemographic and geographical factors, including socioeconomic position and population density, and to compare the current situation (2018/2019) with the year 2006 | 2006, 2018, 2019 | Population: Statistics Netherlands (CBS) (mortality) Meteorological: database of The Royal Netherlands Meteorological Institute | The Netherlands | General population |
| Di Blasi_2023 | Di Blasi et al. (2023) | Effects of temperatures and heatwaves on occupational injuries in the agricultural sector in Italy | To estimate the association between daily air temperatures and occupational injuries in the agricultural sector at the municipal level in Italy and to identify individual vulnerability factors among agricultural workers | 2014-2018 | Population: Italian workers’ compensation authority (INAIL) archives (work-related injuries) Meteorological: ERA-5 Land climate reanalysis data] from the Copernicus Climate data Store (CDS) | Italy | Workers in the agricultural sector |
| Gariazzo_2023 | Gariazzo et al. (2023) | Association between extreme temperature exposure and occupational injuries among construction workers in Italy: An analysis of risk factors | To investigate the association between extreme temperatures and occupation injuries among construction workers, to get an insight in the main accidents-related parameters | 2014-2019 | Population: Italian National Institute for Insurance against Accidents at Work (INAIL) (Occupational Injuries); National Institute of Statistics (ISTAT)  Meteorological: RA5-land Copernicus dataset | Italy | Workers in the construction sector |
| Jahan_2022 | Jahan et al. (2022) | The adverse effect of ambient temperature on respiratory deaths in a high population density area: The case of Malta | To examine the impact of ambient temperature on respiratory deaths in the Maltese population from 1992 to 2017 | January 1992 to December 2017. | Population: Malta’s mortality register (mortality) Meteorological: Malta’s sole Climatological Station situated at Luqa from the global surface archives of WeatherGraphics.com | Malta | General population |
| Kotecki_2023 | Kotecki et al. (2023) | The Impact of Meteorological Parameters and Seasonal Changes on Reporting Patients with Selected Cardiovascular Diseases to Hospital Emergency Departments: A Pilot Study | To determine the meteorological parameters, their interactions, and the seasonal changes of the most significant factors in predicting the number of patients with selected cardiovascular diseases that reported to the EDs in Poznan (Poland) in 2019 | 2019 | Population: National Health Fund (NFZ) database Meteorological: Poznan–Ławica meteorological station | Poland (Poznan) | Patients with selected cardiovascular diseases |
| Márovics_2023 | Márovics et al. (2023) | How vulnerable are patients with COPD to weather extremities? - A pilot study from Hungary | To determine how the extremes of certain meteorological parameters affected the number of visits made by patients with COPD to the local emergency department (ED) | 2017 | Meteorological: monitoring station from the National Oceanic and Atmospheric Administration website; meteorological station operated by the Department of Physical and Environmental Geography, Institute of Geography and Earth Sciences, University of Pécs Faculty of Sciences.  Population: University of Pécs Clinical Center’s e-MedSolution database | Hungary (Pécs) | Patients with COPD aged 18 or older |
| Masselot_2023 | Masselot et al. (2023) | Excess mortality attributed to heat and cold: a health impact assessment study in 854 cities in Europe | To provide a comprehensive and consistent assessment of the current mortality burden associated with non-optimal temperature across most European cities, characterising differences in risks due to local distributions of vulnerability factors and demographic distributions | 2000-2020 | Population: Multi-country Multi-city (MCC) Collaborative Research Network; Eurostat Meteorological: (ERA5)-Land city-specific variables to differentiate patterns of vulnerability across urban populations within Europe from multiple sources | 854 cities from 27 countries in the EU and Norway, Switzerland, and the UK | General population |
| Navas-Martín_2022 | Navas-Martín et al. (2022) | Gender differences in adaptation to heat in Spain (1983–2018) | To analyze the values of minimum mortality temperature in men and women and its temporal evolution during the 1983–2018 period in Spain’s provinces | 1983-2018 | Population: National Statistics Institute (INE) Meteorological: State Meteorological Agency (AEMET) | Spain | General population |
| Navas-Martín_Elderly_2023 | Navas-Martín_1 et al. (2023) | Heat adaptation among the elderly in Spain (1983–2018) | To ascertain elderly persons’ adaptation to heat across the period 1983–2018, through analysis of the minimum mortality temperature and the differences with respect to the total population | 1983-2018 | Population: National Statistics Institute (INE)  Meteorological: State Meteorological Agency (AEMET) | Spain | Population aged ≥ 65 years |
| Navas-Martín_Territory_2023 | Navas-Martín_2 et al. (2023) | Territory differences in adaptation to heat among persons aged 65 years and over in Spain (1983–2018) | To use the time trend in the minimum mortality temperature to ascertain differences in the Spanish elderly population’s adaptation to heat by type of territory | 1983-2018 | Population: National Statistics Institute (INE)  Meteorological: State Meteorological Agency (AEMET) | Spain | Population aged ≥ 65 years |
| Psistaki_2022 | Psistaki et al. (2022) | The impact of ambient temperature on cardiorespiratory mortality in northern Greece | To explore the impact of both high and low ambient temperatures on mortality from cardiorespiratory diseases in a sub-region of the Mediterranean basin, the region of Eastern Macedonia, and Thrace (EMT) in the northeastern part of Greece. | 1999-2018 | Meteorological: three meteorological stations Population: Hellenic Statistical Authority | Greece ( Eastern Macedonia, and Thrace, sub-region of the Mediterranean basin) | General population |
| Revich_2022 | Revich et al. (2022) | The infuence of heat and cold waves on mortality in Russian subarctic cities with varying climates | To assess the excess mortality during heat waves and cold spells identifed during the period 1999–2019 in the Russian subarctic populations and to establish the differences in the exposure to the extreme temperature events that might be related to climate typ | 1999-2019 | Population: Ferderal Statistical (mortality) Meteorological: Russian Institute of Hydrometeorology Information | Russia (Murmansk, Archangelsk, Yakutsk, Magadan) | General population |
| Venturelli_2023 | Venturelli et al. (2023) | High temperature, COVID-19, and mortality excess in the 2022 summer: A cohort study on data from Italian surveillances | To assess whether the effect of high temperature on mortality differed in COVID-19 survivors and naive | 2020-2022 | Population: summer mortality surveillance | Italy (Reggio Emilia province) | General population |
| Vésier_2023 | Vésier et al. (2023) | Gender inequalities in heat-related mortality in the Czech Republic | To quantify heat-related mortality and identify potential sex and gender inequalities in heat vulnerability in the Czech Republic taking into account other factors such as age and marital status/social network | 1995-2019; summer | Population: Institute of Health Information and Statistics of the Czech Republic (UZIS) and the Czech Statistical Office (CZSO) (mortality; SES) Meteorological: Czech Hydrometeorological Institute (CHMI) | Czech Republik | General population |
| Cohort design | | | | | | | |
| Bundo_2023 | Bundo et al. (2023) | How ambient temperature affects mood: an ecological momentary assessment study in Switzerland | To investigate the associations between maximum ambient temperatures and daily bad mood, and identify variables affecting the strength of these associations | Between 2003 and 2006 | Meteorological: urban weather station NABLAU located in the city of Laussane Pollution: Nabel station in the city of Laussane run by the Department of Industrial, Urban and Rural Environment in Vaud (DGE-DIREV) Psychiatric disorders: semi-structured Diagnostic Interview for Genetic Studies (DIGS) Personality trait neuroticism: Eysenck Personality Questionnaire (EPQ) Mood level and sleep quality: Likert scale ranging from one to seven | Switzerland (Lausanne) | General population of Lausanne (follow up of the original sample of 6,734 individuals aged 35 to 75-year-old randomly selected between 2003 and 2006 from the population of the city of Lausanne) |
| Encinas_2023 | Encinas et al. (2023) | Ambient temperature modulates body weight changes in patients with advanced oncological diseases and anorexia cachexia syndrome | To assess the seasonal influence of environmental temperature on the evolution of bodyweight in patients with advanced cancer and anorexia-cachexia syndrome, a wasting syndrome causing dramatic weight loss | 2017 to 2020 | Population: medical records from four hospitals in Extremadura Meteorological: Spanish State Meteorological Agency (AEMET) | Spain (Extremadura) | Patients with a diagnosis of oncological disease in stages 3 or 4 and anorexia- achexia syndrome with weight loss of more than 5% in the last 3 months |
| Guilbert_2023 | Guilbert et al. (2023) | Association of prenatal and postnatal exposures to warm or cold air temperatures with lung function in young infants | To investigate the association of prenatal and postnatal heat or cold exposure with newborn lung function and identify windows of susceptibility | July 2014 - July 2017 | Population: prospective mother-child cohort SEPAGES | France (French Grenoble metropolitan area) | Mother-child cohort |
| Hajat_2023 | Hajat et al. (2023) | Ambient temperature and emergency hospital admissions in people experiencing homelessness: London, United Kingdom, 2011–2019 | To assess the impacts of ambient temperature on hospitalizations of people experiencing homelessness in Greater London | 2011-2019 | Population: Hospital Episode Statistics database by NHS Digital (Emergency hospital inpatient admission)  Meteorological: HadUK-Grid dataset from the Met Office | United Kingdom (London) | Homeless population |
| Hough_2023 | Hough et al. (2023) | Early delivery following chronic and acute ambient temperature exposure: A comprehensive survival approach | To estimate daily temperature exposure throughout pregnancy for three French prospective mother–child cohorts and to evaluate the association between temperature and preterm birth | Three prospective cohort studies:EDED 2003-2006 PELADIE 2002-2006 SEPAGES 2014-2017 | Population: 3 French prospective mother-child cohorts: EDEN (E´ tude des De´terminants pre´ et post natals du de´veloppement et de la sante´ de l’Enfant), 33 PELAGIE (Perturbateurs Endocriniens: e´tude Longitudinale sur les Anomalies de la Grossesse, l’Infertilite´, et l’Enfance)34 and SEPAGES (Suivi de l’Exposition a` la Pollution Atmosphe´rique durant la Grossesse et Effets sur la Sante´) Meteorological: multi-resolution hybrid spatiotemporal model | France (metropolitan areas of Poitiers and Nancy; Brittany region; metropolitan area of Grenoble) | Mother-child cohort |
| Ordanovich_2023 | Ordanovich et al. (2023) | Temporal variation of the temperature-mortality association in Spain: A nationwide analysis | To explore the long-term adaptation to non-optimal temperatures in Spain at the national level by estimating the temporal variations of the minimum mortality temerature and the mortality burden from heat and cold | 1979-2018 | Population: Spain National Institute of Statistics (INE) (vital statistics) Meteorological: European Centre for Medium-Range Weather Forecasts (ECMWF) | Spain | General population |
| Ozturk_2023 | Ozturk et al. (2023) | The effects of heatwaves on hospital admissions in the Edirne province of Turkey - A cohort study | To reveal the effects of HWs on human health in Edirne city (Turkey) based on their age, sex, and chronic diseases, by examining changes in the number of patients in hospital outpatient clinics during the heatwave period | May 1 to September 30, when high temperatures were recorded. Instead of classic summer months period (June- August), we used extended summer months, (May-September) because, temperature records began to be broken frequently in this 5-month period 2018 | Population: Edirne Provincial Health Directorate (hospital data) Meteorological: Turkish State Meteorological Service | Turkey (Edirne province) | General population |
| Roubille_2023 | Roubille et al. (2023) | Impact of global warming on weight in patients with heart failure during the 2019 heatwave in France | To analyse if the body weight of patients with heart failure changes under the pressure of a heatwave | 1.06.2019 - 30.09.2019 | Population: telemonitoring system managed by CDM e-Health, an Air Liquide Healthcare affiliate Meteorological: closest weather station from the residence of each patient within the SYNOP data | France | Patients with chronic heart failure hospitalized for acute heart failure in the previous month and patients hospitalized in the past year with either clinical NYHA Class II or more, or increased natriuretic peptide |
| Savic_2023 | Savić et al. (2023) | Hospital admission tendencies caused by day‑to‑day temperature changes during summer: A case study for the city of Novi Sad (Serbia) | To investigate whether and to what extent sudden changes in temperature, after a change in weather conditions, or the beginning or end of a heat wave, are the cause of the increased number of hospital admissions and what the relationship between the temperature and the tendency of hospital admissions is | 2016-2017 Datasets from the summer seasons (June, July, August) from both years are selected for further analysis. | Meteorological: Novi Sad Urban Network (NSUNET) system  Population: Institute of Public Health of Vojvodina | Serbia (Novi Sad) | General population |
| Yüzen_2023 | Yüzen et al. (2023) | Increased late preterm birth risk and altered uterine blood flow upon exposure to heat stress | To identify the impact of heat exposure on the risk of preterm birth in Hamburg (Germany) | 1999-2021 (March-September) | Population:  central electronic patient registry of the Department of Obstetrics and Fetal Health, University Medical Centre Hamburg-Eppendorf; PRINCE study  Meteorological: federal German National Meteorological Service (Deutscher Wetterdienst) | Germany (Hamburg) | Persons carrying singleton pregnancies |
| Case-control design | | | | | | | |
| Demoury_2022 | Demoury et al. (2022) | Association between temperature and natural mortality in Belgium: Effect modification by individual characteristics and residential environment | To examine the association between extreme temperatures and natural, overall and cause-specific mortality in nine Belgian agglomeration and to investigate the effect modification of this association by individual characteristics | January 1st, 2010 and December 31st, 2015 | Population: Statbel, the Belgian statistical office ( date of death, sex, age (5-year age groups) and cause of death); InterMutualistic Agency (IMA) Pollution: Belgian Interregional Environment Agency Meteorological: Royal Meteorological Institute of Belgium Pollution: Belgian Interregional Environment Agency | Nine largest municipalities and their agglomerations in Belgium | General population |
| Gamboa_2023 | Gamboa et al. (2023) | Analysis of heat stroke and heat exhaustion cases in EudraVigilance pharmacovigilance database | To examine the reported cases of exhaustion-dehydration syndrome (heat exhaustion), or heat stroke associated with any drug notified to the European pharmacovigilance database (EudraVigilance) | January 1, 1995 up to January 10, 2022 | Population: EudraVigilance (European pharmacovigilance database) | European Economic Area | Patients with cases reported to the national drug regulatory agencies (which include cases spontaneously reported by different healthcare professionals) and pharmaceutical companies |
| Guolo_2022 | Guolo et al. (2022) | Emergency department visits and summer temperatures in Bologna, Northern Italy, 2010–2019: A case-crossover study and geographically weighted regression methods | To evaluate the association between summer temperatures and emergency department visits in Bologna (Italy) and assess whether this association varies acrossareas with different socioeconomic and microclimatic characteristics | summers of 2010–2019 | Population: Emergency Department Archive of all Public Health facilities of Bologna Meteorological: Regional Agency for Prevention, Environment and Energy of Emilia Romagna | Italy (Bologna) | Population, who had at least one emergency department visit in the period 2010–2019 during the summer season from 1 June to 30 September |
| Hinchliffe_Alguacil_2023 | Hinchliffe; Alguacil et al. (2023) | Occupational heat exposure and prostate cancer risk: A pooled analysis of case-control studies | To analyse potential associations between occupational heat exposure and prostate cancer risk in three different countries and to investigate possible interactions between occupational heat exposure and other occupational agents | PROtEuS (Prostate Cancer & Environment Study) 2005-2012 MCC-Spain 2008-2014 EPICAP (Epidemiological Study of Prostate Cancer) 2012-2014 | Population: PROtEuS (Prostate Cancer & Environment Study); MCC-Spain; EPICAP (Epidemiological Study of Prostate Cancer) | Canada (Montreal metropolitan area), France (H´erault region), Spain (seven regions) | Canada: Citizens registered on the provincial electoral list and aged <76 years at diagnosis or interview.  Spain: Participants aged 40–85 years, residence in the catchment area for ≥6 months prior to recruitment and capable of answering the epidemiological questionnaire. France: Patients newly diagnosed with prostate cancer in 2012–2013 and <75 years old |
| Hinchliffe_Kogevinas_2023 | Hinchliffe; Kogevinas et al. (2023) | Association of occupational heat exposure and colorectal cancer in the MCC-Spain study | To analyse the potential association of life-time occupational heat exposure and colorectal cancer risk | 2008-2013 | Population: MCC-Spain | Spain (12 administrative regions) | Population aged 20–85 years, residence in the catchment area for ≥6 months prior to recruitment, no prior history of CRC and ability to answer the epidemiological questionnaire |
| Ragettli_2023 | Ragettli et al. (2023) | Explorative assessment of the temperature–mortality association to support health-based heat-warning thresholds: A national case-crossover study in Switzerland | To investigate the relationship between temperature and mortality in Switzerland | Warm season (May to September) between 2003 and 2016 | Population: Swiss National Cohort (SNC) (mortality)  Meteorological: temperature model by Flückiger et al. | Switzerland | General population |
| Yarza_2023 | Yarza et al. (2023) | Ability to adapt to seasonal temperature extremes among atrial fibrillation patients. A nation-wide study of hospitalizations in Israel | To investigate the relationship between hospitalizations due to atrial fibrillation and exposure to ambient temperature | 2004-2018 | Population: seven hospitals within Clalit Health Services  Meteorological: satellite-based model proposed by Zhou et al. (2020) | Israel | Patients >18, diagnosed with atrial fibrillation |
| Cross-sectional design | | | | | | | |
| Darabi_2023 | Darabi et al. (2023) | Environmental stress, minority status, and local poverty: Risk factors for mental health in Berlin’s inner city | To measure associations between local poverty and environmental factors and asses whether the effects of local poverty on mental health are mediated by local heat exposure, air pollution, noise pollution, and greenspace exposure | 2009-2011 | Meteorological: Flow over Irregular Terrain with Natural and Anthropogenic Heat sources (FITNAH) climate model and long-standing measuring stations in Berlin and Potsdam; green space information system (GRIS) Population: Berlin Senate (Department for Urban Development, Building and Housing) and the Berlin-Brandenburg Office for Statistics; public registries of all residents in eleven neighborhoods in Berlin’s inner city (Berlin-Mitte), interviews and General Health Questionnaire 28-item version (GHQ-28) Pollution: Environmental Justice Atlas from the Berlin Senate Administration for Urban Development, Building and Housing (environmental stress factors); data from 400 detectors in 300 different locations throughout Berlin’s main street ( NO2 and PM2,5 emissions cars); Federal Environmental Agency’s  handbook for emission factors (all transportations) | Germany (Berlin) | Population between 18 to 68 years |
| Zeren_2023 | Zeren Cetin et al. (2023) | A geographic information systems and remote sensing–based approach to assess urban micro‑climate change and its impact on human health in Bartin, Turkey | To inspect the impact of urban microclimate change, i.e., increasing land surface temperature, on human health in Bartin (Turkey) | March 2000 and March 2020 for satellite data, interviews May and June 2020-21 | Meteorological: Landsat 7 ETM satellite dataset for the year 2000 (March 7th) and Landsat 8 OLI (Operation Land Imager) and TIRS (Thermal Infrared Sensor) satellite dataset for the year 2020 (March 8th)  Population: Interviews | Turkey (Bartin) | General population |
| Qualitative design | | | | | | | |
| Brooks_2023 | Brooks et al. (2023) | Heatwaves, hospitals and health system resilience in England: A qualitative assessment of frontline perspectives from the hot summer of 2019 | To assess the impacts of very hot weather on (i) frontline staff in hospitals in England and (ii) on healthcare delivery and patient safety | "between October 2019 and January 2020" | Preinterview survey and semi-structured interviews | England | Health professionals in the National Health Service (clinicians and non-clinicians, including facilities managers and emergency preparedness, resilience and response professionals) |

Appendix 6: Results of searches for primary studies

Description of included studies

The search yielded 726 unique results. Overall, 99 primary studies were included for full text review after title and abstract screening and 41 primary studies were included for further analysis.

Study design

Among the 41 included studies, we found five categories of study design: Ecological (n=21), cohort studies (prospective or retrospective) (n=12), case-control studies (with or without case-crossover analysis) (n=7), cross-sectional studies (n=2) and qualitative studies (n=1).

Geographic focus

Three studies included data from several European countries [82-84] and one included data from countries worldwide[85]. One study focused on one or more regions from three countries (Canada, France, Spain)[86]. Most included studies, however, focused on one country (n=16) or one city (n=9). Ten studies were concerned with one or more regions in a country. One study covered four Russian cities[87].

Population

Slightly less than half of the included primary studies indicated the ‘general population’ as their population of interest (n=23). The other studies focused on populations with various pre-existing health conditions [84, 88-92] or populations above a certain age e.g. 65 or higher[93-95]. In further studies, the populations of interest were workers[96, 97], mothers and their children[98, 99], singleton pregnancies[100], persons attending emergency departments[101], health professionals[102], and persons experiencing homelessness[103]. One study reported outcomes from a combination of different populations, including the general population within a certain age range and patients newly diagnosed with prostate cancer[86].

Mapping of inequalities

Most studies included disaggregated outcomes for age (n=31) and sex/gender (n=26) (Table 3). Thirteen studies disaggregated outcomes for pre-existing health conditions. Of these none was explicitly concerned with (dis)ability and only one focused explicitly on alcohol consumption[102]. Disaggregated outcomes for area of living / housing conditions were reported in eleven primary studies but only two of those considered nursing homes or health care facilities [102, 104] and only one homelessness[103].

Outcomes were disaggregated for compound indicators of socioeconomic status [88, 93, 101, 105-107] or work determinants in six studies[86, 96, 97, 102, 108, 109]. For other determinants of inequalities, disaggregated health outcomes were included in four or fewer studies.

Disaggregated health outcomes within different population groups were assessed in few studies, e.g. outcomes in urban versus rural populations for sex, age and health conditions (here, circulatory system disease)[110].

Table 3 - Determinants of inequalities considered in primary studies

|  | **Determinants of inequalities considered in primary studies** | | | | | | | | | | | |
| --- | --- | --- | --- | --- | --- | --- | --- | --- | --- | --- | --- | --- |
| **Study ID** | **Sex / gender** | **Age** | **Marital status/social network** | **Ethnicity** | **Education** | **Income** | **Work*** | **Area of living / housing conditions** | **Ecological parameters** | **Health condition including ability** | **SES** | **Other** |
| **Ecological studies** | | | | | | | | | | | | |
| Astone_2022 | nr | yes | nr | nr | nr | nr | nr | nr | nr | yes | yes | nr |
| Ballester_2023 | yes | yes | nr | nr | nr | nr | nr | nr | nr | nr | nr | nr |
| Chitu_2023 | yes | yes | nr | nr | nr | nr | nr | yes | nr | yes | nr | nr |
| Choi_2022 | nr | nr | nr | nr | nr | nr | nr | nr | yes | nr | nr | nr |
| Coma_2023 | yes | yes | nr | nr | nr | nr | nr | nr | nr | nr | nr | nr |
| Conte_2022 | yes | yes | yes | nr | nr | nr | nr | nr | nr | nr | yes | nr |
| de Schrijver_2023 | nr | yes | nr | yes | nr | nr | nr | yes | yes | nr | yes | nr |
| de Visser_2023 | yes | yes | nr | nr | nr | yes | yes | yes | nr | nr | nr | nr |
| Di Blasi_2023 | yes | yes | nr | nr | nr | nr | yes | nr | nr | nr | nr | nr |
| Gariazzo_2023 | nr | yes | nr | nr | nr | nr | yes | nr | nr | nr | nr | nr |
| Jahan_2022 | yes | yes | nr | nr | nr | nr | nr | nr | nr | nr | nr | nr |
| Kotecki_2023^1^ | nr | nr | nr | nr | nr | nr | nr | nr | nr | yes | nr | nr |
| Márovics_2023^1^ | nr | nr | nr | nr | nr | nr | nr | nr | nr | yes | nr | nr |
| Masselot_2023 | nr | yes | nr | nr | nr | nr | nr | nr | nr | nr | nr | nr |
| Navas-Martín_2022 | yes | nr | nr | nr | nr | nr | nr | nr | nr | nr | nr | nr |
| Navas-Martín_Elderly_2023 | nr | yes | nr | nr | nr | nr | nr | nr | nr | nr | nr | nr |
| Navas-Martín_Territory_2023 | nr | yes | nr | nr | nr | nr | nr | yes | nr | nr | nr | nr |
| Psistaki_2022 | yes | yes | nr | nr | nr | nr | nr | nr | nr | nr | nr | nr |
| Revich_2022 | nr | yes | nr | nr | nr | nr | nr | nr | nr | nr | nr | nr |
| Venturelli_2023 | yes | yes | nr | nr | nr | nr | nr | yes | nr | yes | nr | nr |
| Vésier_2023 | yes | yes | yes | nr | nr | nr | nr | nr | nr | nr | nr | nr |
| **Cohort studies** | | | | | | | | | | | | |
| Bundo_2023 | nr | yes | yes | nr | yes | nr | nr | nr | nr | yes | nr | nr |
| Encinas_2023 | yes | yes | nr | nr | nr | nr | nr | yes | nr | yes | nr | nr |
| Guilbert_2023 | yes | nr | nr | nr | nr | nr | nr | nr | nr | nr | nr | nr |
| Hajat_2023^1^ | nr | nr | nr | nr | nr | nr | nr | yes | nr | nr | nr | nr |
| Hough_2023^1^ | yes | nr | nr | nr | nr | nr | nr | nr | nr | nr | nr | nr |
| Ordanovich_2023 | yes | yes | nr | nr | nr | nr | nr | nr | nr | nr | nr | nr |
| Ozturk_2023 | yes | yes | nr | nr | nr | nr | nr | nr | nr | yes | nr | nr |
| Roubille_2023 | yes | yes | nr | nr | nr | nr | nr | nr | nr | yes | nr | nr |
| Savic_2023 | nr | yes | nr | nr | nr | nr | nr | nr | nr | nr | nr | nr |
| Yüzen_2023 | yes | yes | nr | nr | nr | nr | nr | nr | nr | nr | nr | yes |
| **Case-control studies** | | | | | | | | | | | | |
| Demoury_2022 | yes | yes | nr | nr | nr | nr | yes | yes | yes | yes | nr | nr |
| Gamboa_2023 | yes | yes | nr | nr | nr | nr | nr | nr | nr | nr | nr | nr |
| Guolo_2022 | yes | yes | nr | nr | nr | yes | nr | nr | nr | nr | yes | nr |
| Hinchliffe_Alguacil_2023 | nr | nr | nr | nr | nr | nr | yes | nr | nr | yes | nr | yes |
| Hinchliffe_Kogevinas_2023 | yes | nr | nr | nr | yes | nr | nr | nr | nr | yes | nr | yes |
| Ragettli_2023 | yes | yes | nr | nr | nr | nr | nr | nr | nr | nr | nr | nr |
| Yarza_2023 | yes | yes | nr | yes | nr | nr | nr | nr | nr | nr | yes | nr |
| **Cross-sectional studies** | | | | | | | | | | | | |
| Darabi_2023 | yes | yes | nr | yes | yes | yes | nr | yes | yes | nr | yes | nr |
| Zeren_2023 | nr | nr | nr | nr | nr | yes | nr | yes | nr | nr | nr | nr |
| **Qualitative studies** | | | | | | | | | | | | |
| Brooks_2023 | yes | yes | nr | nr | nr | nr | yes | yes | nr | yes | nr | nr |

**Legend**: nr = not reported, *work including employment status, (un)employment rate, working environment, ^1^These studies only reported outcomes for one subpopulation (with no comparison).
